# TryCYCLE: A Prospective Study of the Safety and Feasibility of Early In-Bed Cycling in Mechanically Ventilated Patients

**DOI:** 10.1371/journal.pone.0167561

**Published:** 2016-12-28

**Authors:** Michelle E. Kho, Alexander J. Molloy, France J. Clarke, Daana Ajami, Magda McCaughan, Kristy Obrovac, Christina Murphy, Laura Camposilvan, Margaret S. Herridge, Karen K. Y. Koo, Jill Rudkowski, Andrew J. E. Seely, Jennifer M. Zanni, Marina Mourtzakis, Thomas Piraino, Deborah J. Cook, Canadian Critical Care Trials Group

**Affiliations:** 1 School of Rehabilitation Science, McMaster University, Hamilton, Ontario, Canada; 2 Department of Physiotherapy, St. Joseph’s Healthcare, Hamilton, Ontario, Canada; 3 Department of Physical Medicine and Rehabilitation, Johns Hopkins University, Baltimore, Maryland; 4 Department of Clinical Epidemiology and Biostatistics, McMaster University, Hamilton, Ontario, Canada; 5 Department of Medicine, University of Toronto, Toronto General Research Institute, University Health Network, Toronto, Ontario, Canada; 6 Swedish Early Mobility Program in Critical Care, First Hill Campus, Swedish Medical Group, Seattle, Washington; 7 Department of Medicine, Western University, London, Ontario, Canada; 8 Department of Medicine, McMaster University, Hamilton, Ontario, Canada; 9 Ottawa Hospital Research Institute, University of Ottawa, Ottawa, Ontario, Canada; 10 Department of Kinesiology, University of Waterloo, Waterloo, Ontario, Canada; 11 Respiratory Therapy Service, St. Joseph’s Healthcare, Hamilton, Ontario, Canada; Universidade do Extremo Sul Catarinense, BRAZIL

## Abstract

**Introduction:**

The objective of this study was to assess the safety and feasibility of in-bed cycling started within the first 4 days of mechanical ventilation (MV) to inform a future randomized clinical trial.

**Methods:**

We conducted a 33-patient prospective cohort study in a 21-bed adult academic medical-surgical intensive care unit (ICU) in Hamilton, ON, Canada. We included adult patients (≥ 18 years) receiving MV who walked independently pre-ICU. Our intervention was 30 minutes of in-bed supine cycling 6 days/week in the ICU. Our primary outcome was Safety (termination), measured as events prompting cycling termination; secondary Safety (disconnection or dislodgement) outcomes included catheter/tube dislodgements. Feasibility was measured as consent rate and fidelity to intervention. For our primary outcome, we calculated the binary proportion and 95% confidence interval (CI).

**Results:**

From 10/2013-8/2014, we obtained consent from 34 of 37 patients approached (91.9%), 33 of whom received in-bed cycling. Of those who cycled, 16(48.4%) were female, the mean (SD) age was 65.8(12.2) years, and APACHE II score was 24.3(6.7); 29(87.9%) had medical admitting diagnoses. Cycling termination was infrequent (2.0%, 95% CI: 0.8%-4.9%) and no device dislodgements occurred. Cycling began a median [IQR] of 3 [2, 4] days after ICU admission; patients received 5 [3, 8] cycling sessions with a median duration of 30.7 [21.6, 30.8] minutes per session. During 205 total cycling sessions, patients were receiving invasive MV (150 [73.1%]), vasopressors (6 [2.9%]), sedative or analgesic infusions (77 [37.6%]) and dialysis (4 [2.0%]).

**Conclusions:**

Early cycling within the first 4 days of MV among hemodynamically stable patients is safe and feasible. Research to evaluate the effect of early cycling on patient function is warranted.

**Trial Registration:**

Clinicaltrials.gov: NCT01885442

## Introduction

Functional disability can last for many years in critical illness survivors [1,2]. Due to an aging population, and increasing survival from critical illness [3,4], the burden of physical and cognitive disability among patients discharged from the intensive care unit (ICU) is also increasing [5]. A systematic review of 14 randomized clinical trials (RCTs) identified that exercise-based physical therapy (PT) interventions started in the ICU were most effective to improve physical function compared to other strategies such as nutrition or different modes of mechanical ventilation (MV) [6]. This improved function following critical illness may be due to addressing the early and rapid reduction in muscle size and strength that occur within the first 10 days of a patient’s ICU stay [7,8]. Thus, interventions to prevent or reduce muscle size and minimize strength losses within this early time period may help to improve long-term outcomes in ICU survivors.

Rehabilitation interventions started very early in a patient’s ICU stay can improve function at hospital discharge. In a 104-patient RCT, those who received occupational (OT) and PT interventions in the ICU within 1.5 days of starting MV were more likely to be functionally independent at hospital discharge than those started at 7.4 days [9]. Here, the main difference was receipt of 20 minutes of therapy during MV by the intervention group versus no therapy during MV by the control group. However, common ICU interventions like MV can pose barriers to rehabilitation. For example, a recent prospective cohort study reported that the presence of an oral endotracheal tube (ETT) was an important barrier to receipt of mobilization within the first 14 days of MV [10].

In-bed cycling (“cycling”) is a promising early intervention for critically ill patients, with evidence supporting its use later in a patient’s ICU stay. In a 90-patient RCT, those receiving cycling started 14 days after ICU admission versus usual care had better 6-minute walk (6 MWT) distances, greater leg strength, and better Short Form 36 (SF-36) physical function scores at hospital discharge [11]. Commercially-available devices can provide 3 possible activity modes: passive (i.e., fully motorized, no patient initiation), active-assisted (i.e., partially initiated by the patient), or active (i.e., fully initiated by the patient). Cycling can enhance rehabilitation in critically ill patients[12] by providing low-intensity movement, allowing patients’ spontaneous participation in activity, and facilitating rest breaks in severely deconditioned patients.

Cycling started earlier in a patient’s ICU stay may further improve patient outcomes. However, evidence for early cycling is limited to observations of single sessions [13], cycling incorporated in a multi-modal rehabilitation strategy [14,15], a case-control study of cycling with functional electrical stimulation[16], or retrospective review of cycling in routine PT care [17]. In preparation for a larger trial of this intervention, the objective of this study was to evaluate the safety and feasibility of early leg cycling in critically ill patients.

## Materials and Methods

We enrolled adult patients (>18 years) from a 21-bed academic medical-surgical ICU in Hamilton, ON, Canada. Immediately upon ICU admission, a research coordinator screened for eligible patients: MV for 0 to ≤4 days, ICU LOS for 0 to ≤7 days, and who were able to ambulate with or without a gait aid before hospitalization. Pre-enrolment, we screened patients for the duration of their eligibility as long as they met the MV and LOS criteria. Table 1 outlines study exclusion criteria and cycling exemptions developed from a systematic review of early mobility activities [18], clinical trials published at the time of study design [9,11,19], and clinical research team consensus. Written informed consent was obtained by a research coordinator from all participants (or their proxy) included in this open-label study.

**Table 1:** Study Inclusion, Exclusion, and Temporary exemption criteria.

**Inclusion Criteria**
Mechanically ventilated for 0 to ≤4 days
ICU length of stay for 0 to ≤7 days
Able to ambulate with or without a gait aid before hospitalization
**Exclusion Criteria**
Unable to follow commands in English at baseline
Acute condition impairing patient’s ability to cycle (e.g., leg fracture)
Neuromuscular weakness affecting the legs (e.g., stroke, Guillain Barre syndrome)
Temporary pacemaker
Expected hospital mortality >90%
Body habitus unable to fit the bike
Pregnancy
Palliative goals of care
Cycling exemptions precluding cycling within the first 4 days of MV
**Temporary cycling exemptions**
Respiratory
Persistent O_2_ saturation <88%
Cardiovascular
Active myocardial ischemia (MI)
Unstable or uncontrolled arrhythmia
Any increase in vasoactive infusions within the last 4 hours
Mean arterial pressure (MAP) <60 or >110 mmHg within the last 2 hours
Heart rate (HR) <40 or >140 beats per minute (BPM) within the last 2 hours
Receipt of neuromuscular blocking agents within the last 4 hours
Uncontrolled pain
Severe agitation (Richmond Agitation and Sedation Scale, RASS[48]) >2) within the last 2 hours
Change in goals to palliative care
Presence of a femoral arterial or venous catheter*
Team perception that cycling was not appropriate, despite absence of above exemption criteria

This table outlines trial inclusion, exclusion, and temporary cycling exemption criteria. We excluded patients if they had persistent temporary cycling exemption criteria within the first 4 days of mechanical ventilation. Once enrolled, we reviewed patients’ clinical status for temporary cycling exemption criteria daily.

*We removed this temporary exemption following published evidence for the safety of rehabilitation activities for femoral catheter in situ.

### Intervention

We prescribed 30 minutes of leg cycling with an additional 1 minute cool down, 6 days/week, for the duration of the patient’s ICU stay to a maximum of 28 days. This intervention was in addition to routine PT. If a patient was re-admitted to ICU during the index hospitalization, we re-started cycling. We reviewed patients daily for temporary cycling exemptions (Table 1).

**Fig 1:**
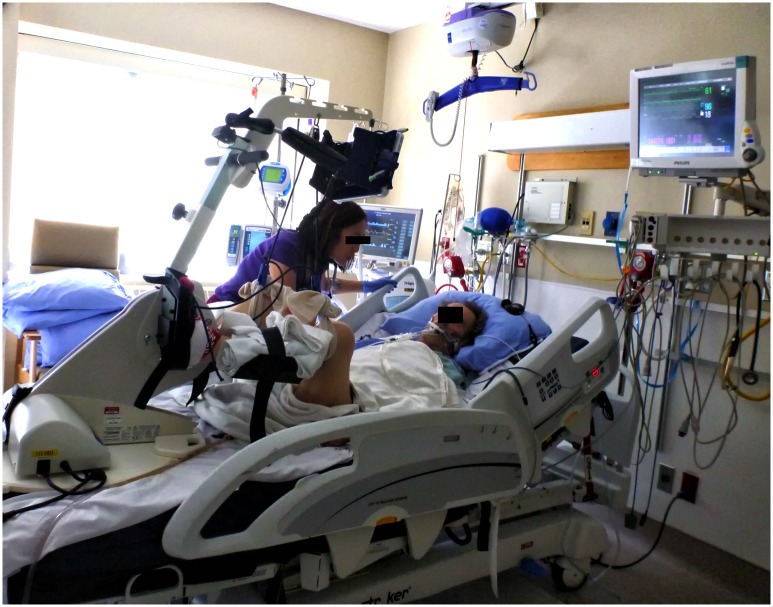
Example of in-bed cycling. This figure demonstrates a patient in the ICU receiving in-bed cycling and mechanical ventilation. An ICU physiotherapist supervises the in-bed cycling session.

ICU PTs led all cycling sessions. We used a specialized cycle ergometer purchased by our hospital (RT300 supine cycle, Restorative Therapies, Baltimore, MD; Fig 1)[11]. All PTs received a 1-day in-service and had over 6 months of clinical experience with the cycle before starting the study. The participants started with passive cycling at a rate of 5 revolutions/minute (RPM) based on our clinical experience where patients actively cycled at low pedal cadence. If patients initiated active cycling, the PT promoted active participation. The therapists encouraged as much active cycling as possible, and low resistance (0.5 NM) was used during active cycling. Due to a variable level of consciousness throughout their stay, we allowed patients to cycle at a self-selected rate. We chose not to add resistance during the cycling intervention because we would not be able to discern if early cycling at a patient’s self-selected pace or increased resistance influenced outcomes in a future RCT. If the patient stopped cycling actively, the ergometer reverted to passive cycling.

We measured vital signs pre-, during (at 5, 10, 20, and 30 minutes), and post- cycling. During cycling, if the patient had 2 consecutive readings of mean arterial pressure (MAP) <60 or >110 mmHg, heart rate (HR) < 40 or >140 beats per minute (BPM), or SpO_2_ <88%, despite adjustments to FiO_2_, we advised PTs to use clinical judgment to stop a cycling session, according to each patient’s individual clinical circumstances in consultation with the ICU team.

We collected the following data: APACHE II score,[20] Charlson Comorbidity Index,[21] and Functional Comorbidity Index [22]. We collected the Katz Activity of Daily Living (ADL) Scale,[23] and Functional Status Score for ICU (FSS-ICU) [24] at study entry from interviews with the proxy or patient. Daily ICU data included Multiple Organ Dysfunction Score (MODS),[25] and exposures including MV, receipt of neuromuscular blockers, vasopressors or inotropes; benzodiazepines, opioids, or propofol; and dialysis. We collected MV, drug exposures and receipt of dialysis as binary variables for each study day.

### Primary Outcome and Sample Size Calculation

Our primary outcome was Safety (termination), defined as receipt of 30-minute leg cycling sessions without stopping due to 5 *a-priori* reasons: 1) unplanned extubation, 2) suspected new unstable or uncontrolled arrhythmia, 3) concern for myocardial infarction (MI), 4) ICU physician request to terminate session, and 5) PT terminated session due to physiologic concerns. We hypothesized that the observed Safety (termination) event rate would not differ, or would be better (i.e., lower) than other early rehabilitation studies (0 to 4%).[18] We estimated we needed 164 cycling sessions to ensure an observed Safety (termination) rate was within an upper 95% confidence interval (CI) of 3% from a point estimate of 4% (7 events) [26]. Assuming a median ICU length of stay of 7 days, 2 days to enroll patients from ICU admission, and 5 cycling sessions per patient, we enrolled 33 patients.

### Secondary Outcomes

#### Safety (disconnection or dislodgement)

Inadvertent ventilator disconnection or device dislodgement (catheters: peripheral venous, arterial, central venous, pulmonary artery, or dialysis; tubes: orogastric, nasogastric, or percutaneous gastrostomy or jejunotomy).

#### Feasibility

Consent rate >70%[9], ability to provide cycling sessions, and ability to collect physical outcome measures at ICU awakening, ICU discharge, and hospital discharge. We recorded the duration of cycling by the patient and the total duration (including patient setup and equipment take-down) of all sessions. Other outcomes included PT assessment of muscle strength (Medical Research Council sum score (MRC-SS),[27,28], ICU-acquired weakness (MRC-SS <48)[29], hand grip[28,30], quadriceps strength with dynamometry[31], and function (FSS-ICU[24], Physical Function ICU Test-scored (PFIT-s)[32,33], 6MWT[34]). We also collected duration of MV, discharge location, ICU and hospital length of stay (LOS) and mortality.

### Analysis

For binary variables, we calculated the binary proportion and 95% CI. For continuous variables, we calculated the mean and standard deviation (SD), or if non-normally distributed, the median and interquartile range [IQR]. We compared continuous variables using a two-sided paired or independent Student’s t-test, as appropriate. We used SAS version 9.2 (Cary, North Carolina) for all analyses and considered p-values ≤0.05 significant.

### Ethical Approval

The Hamilton Integrated Research Ethics Board approved this study (13–173; Clinicaltrials.gov: NCT01885442).

We reported our study according to the Transparent Reporting of Evaluations with Nonrandomized Designs (TREND) Statement[35] and Template for Intervention Description and Replication (TIDieR)[36] checklist (S1 and S2 Tables).

## Results

**Fig 2:**
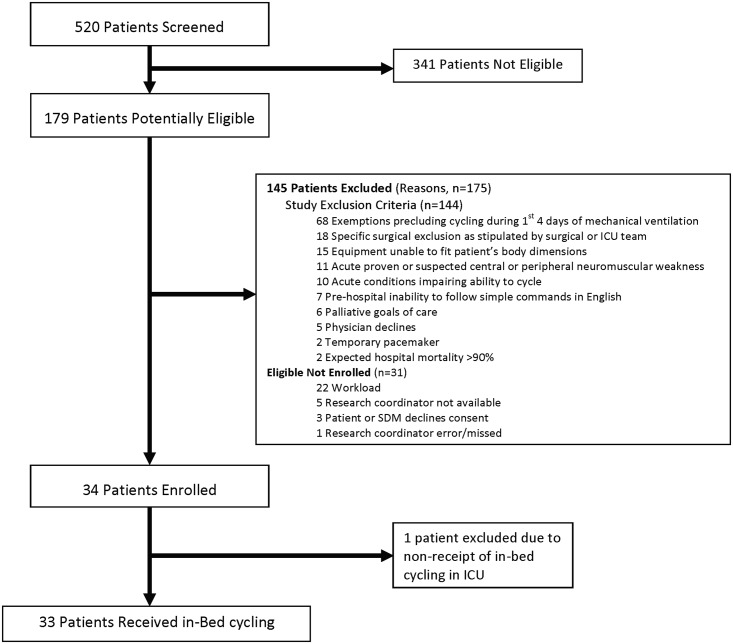
Patient flow diagram. This figure outlines patient screening and enrollment in the TryCYCLE study. The 68 persisting temporary exemptions within the first 4 days of mechanical ventilation included: receipt of neuromuscular blocking agents (n = 19), increase in vasoactive infusions (n = 14), femoral arterial or venous catheter in situ (n = 13), active myocardial infarction of unstable/ uncontrolled arrhythmia (n = 8), severe agitation (n = 2), persistent SpO2 <88% (n = 2), mean arterial pressure <60 mmHg or >110 mmHg (n = 1), heart rate <40 or >140 beats per minute (n = 1), other concern (n = 8).

Between October 30, 2013 and August 18, 2014, we enrolled 34 patients (Fig 2). One patient did not receive any cycling during their ICU stay due to persistently high MAP and was excluded from further analysis. Of all patients, 11 (33.0%) received vasopressors or inotropes, 5 (15.1%) received neuromuscular blockers, and 7 (21.2%) received dialysis during their ICU stay. Table 2 outlines patient characteristics.

**Table 2:** Patient Demographics, Baseline Characteristics, and Outcomes.

Characteristic	N = 33 Patients
Age in years, mean (SD)	65.8 (12.2)
Females, n (%)	16 (48.5)
Race, n (%)	
White	29 (87.9)
Southeast Indian	2 (6.1)
Black	1 (3.0)
Asian	1 (3.0)
Medical admission, n (%)	29 (87.9)
APACHE II[20], mean (SD)	24.3 (6.7)
Admission diagnosis, n (%)	
Respiratory	19 (57.6)
Sepsis	4 (12.1)
Gastrointestinal (non-surgical)	2 (6.1)
Gastrointestinal (surgical)	2 (6.1)
Cardiovascular/vascular	2 (6.1)
Other surgical	2 (6.1)
Renal	1 (3.0)
Other medical	1 (3.0)
Charlson Comorbidity Index[21], mean (SD)	2.2 (2.0)
Functional Comorbidity Index[22], mean (SD)	2.3 (1.4)
Pre-ICU Katz ADL score[23], mean (SD)	5.5 (1.3)
Pre-ICU Functional Status Score for ICU[24], mean (SD)	33.9 (3.2)
Location in hospital before ICU admission, n (%)	
Medical or surgical stepdown	9 (27.3)
Other hospital	8 (24.3)
Emergency department	6 (18.2)
Hospital Ward	6 (18.2)
Operating room/ post-operative recovery room	4 (12.1)
Duration of mechanical ventilation (index admission), median [IQR], days	8 [6, 14]
ICU LOS, median [IQR], days	11 [7, 17]
ICU mortality, n (%)	5 (15)
Hospital LOS, median [IQR], days	31 [16, 42]
Hospital mortality, n (%)	10 (30)
Hospital discharge disposition of 23 survivors, n (%)	
Home, independent	7 (30)
Home, with home care	6 (26)
Repatriated to another hospital	4 (17)
Inpatient rehabilitation	3 (13)
Assisted living facility	1 (5)
Other	2 (9)

This table summarizes patient demographics, baseline characteristics, and patient outcomes. Abbreviations: SD = standard deviation; n = number; LOS = length of stay; APACHE II = Acute Physiology and Chronic Health Evaluation, an 13 item instrument with scores from 0 to 71, higher scores representing higher severity of illness[20]; Charlson Comorbidity Index includes 19 categories of comorbidity, with higher scores representing more comorbidity[21]; Functional Comorbidity Index includes 18 items associated with physical function, with higher scores representing higher comorbid illness[22]; ADL = activities of daily living; Katz score is a 6-item instrument assessing independence in bathing, dressing toileting, transferring, continence, and feeding, with higher scores representing more independence[23].

### Safety

The Safety (termination) rate was 2% (95% CI (0.8% to 4.9%) in 205 cycling sessions (4 events: high MAP (n = 2), SpO_2_ <88% (n = 1), and physician request for termination due to concern for MI (n = 1; subsequent workup revealed no evidence of MI)). There were no unplanned extubations, or Safety (disconnection or dislodgement) events. Of 205 cycling sessions, provided by 5 different PTs, there were 56 respiratory or cardiovascular physiologic changes from baseline (Table 3). In most instances, PTs did not stop cycling early due to these transient changes. There was a statistically, but not clinically significant difference in pre- and post- cycling HR and MAP, and no differences in the remaining vital signs (BP, SpO_2_, FiO_2;_ S3 Table).

**Table 3:** Characteristics of *a-priori* physiologic changes from baseline during in-bed cycling sessions.

Physiologic changes during cycling sessions, n (%)	N = 56
**Respiratory**, n (%)	1(1.8)
Sustained O_2_ desaturation <88%, despite adjustments to FiO_2_	1(1.8)
Marked ventilator dysynchrony, despite adjustments	0
Respiratory distress leading to symptoms of marked dyspnea	0
Unplanned extubation^a^	0
**Cardiovascular**, n (%)	55 (98.2)
High systolic BP: 20 mmHg more than highest baseline value	29 (51.8)
MAP >110 mmHg (non-sustained)	22 (39.3)
High diastolic BP: 20 mmHg more than highest baseline value	8 (14.3)
Low HR: 20 bpm less than baseline value or 40 bpm (highest)	4 (7.1)
Low systolic BP: 20 mmHg less than lowest baseline value	4 (7.1)
High HR: 20 bpm more than highest baseline value or 140 bpm (lowest)	3 (5.4)
Low diastolic BP: 20 mmHg less than lowest baseline value	2 (3.6)
MAP <60 mmHg	0
Suspected new unstable/uncontrolled arrhythmia^a^	0
Concern for myocardial ischemia^a^	0

Data in this table represent a-priori physiologic changes from baseline. Abbreviations: BP = blood pressure; MAP = mean arterial pressure; HR = heart rate; bpm = beats per minute.

^a^*A-priori* Physiologic event leading to immediate termination of in-bed cycling.

### Feasibility

Our consent rate was 91.9% (34/37), and median [IQR] time from ICU admission to consent was 2 [1, 3] days. The median [IQR] time from ICU admission to first cycling was 3 [2, 4] days. Patients received a median [IQR] of 5 [3, 8] sessions, and the duration of cycling and total session (including set up and take down) was 30.2 [20.0, 30.7] and 43 [36, 49] minutes, respectively. Of 320 opportunities, 115 (35.9%) sessions were withheld (Table 4) and patients completed the full 30-minute protocol on 138 of 205 (67.3%) occasions. Table 4 outlines details for the 67 sessions stopped early.

**Table 4:** Summary of reasons for not cycling^a^ or cycling stopped early.

Reasons for not cycling^a^ in 115 sessions	n (%)
**Medical Conditions**	N = 89 (77.3)
Neuromuscular blocker within last 4 hours	37 (32.2)
Mean Arterial Pressure <60 or >110 mmHg within the last 2 hours	22 (19.1)
Team perception that in-bed cycling is not appropriate despite absence of explicit reasons	16 (13.9)
Any increase in vasopressor/ inotrope within last 4 hours	5 (4.3)
Femoral arterial or venous catheter	3 (2.6)
Heart Rate <40 or >140 bpm within the last 2 hours	2 (1.7)
Severe agitation (RASS >2 [or equivalent]) within last 2 hours	2 (1.7)
Change in goals to palliative care	2 (1.7)
Active myocardial ischemia, or unstable/ uncontrolled arrhythmia	1 (0.9)
Persistent SpO_2_ <88% within the last 2 hours	0
Uncontrolled pain	0
**Other Reasons**	N = 32 (27.8)
Patient refused^b^	27 (23.5)
Patient not available (patient out of the ICU)^c^	3 (2.6)
Other^d^	2 (1.7)
**Reasons for cycling stopped early in 67 sessions**	N (%)
Patient request to stop due to fatigue	48 (71.6)
Patient agitation	5 (7.4)
*A priori* safety (termination) concerns	4 (6.0)
Perceived patient discomfort	3 (4.5)
Bowel movement during cycling	2 (3.0)
Peripheral intravenous foot catheter interfering with cycling motion	1 (1.5)
Cycle ergometer malfunction	1 (1.5)

This table summarizes reasons for not cycling that were recorded during daily review pre-cycling and reasons for stopping cycling early (i.e., before 30 minutes). Abbreviations: RASS = Richmond Agitation and Sedation Scale; bpm = beats per minute; ICU = intensive care unit.

^a^Totals sum greater than 115 because each session could have more than one reason for not cycling. Data are reasons as a proportion of 115 sessions.

^b^Of 27 sessions, 10 patients refused 1 or more cycling sessions, and 2 patients accounted for 16 (60%) of all refusals (10 and 6 refusals each). On 6 of these occasions, patients received alternate mobility activities (e.g., sitting at the edge of the bed, sitting in a chair) on the same day.

^c^Patients were not available due to procedures in the operating room (n = 2) or diagnostic imaging (n = 1).

^d^Other includes bike unable to fit on bed (n = 1), left foot intravenous catheter interfering with cycling (n = 1).

### Cycling Session Characteristics

**Fig 3:**
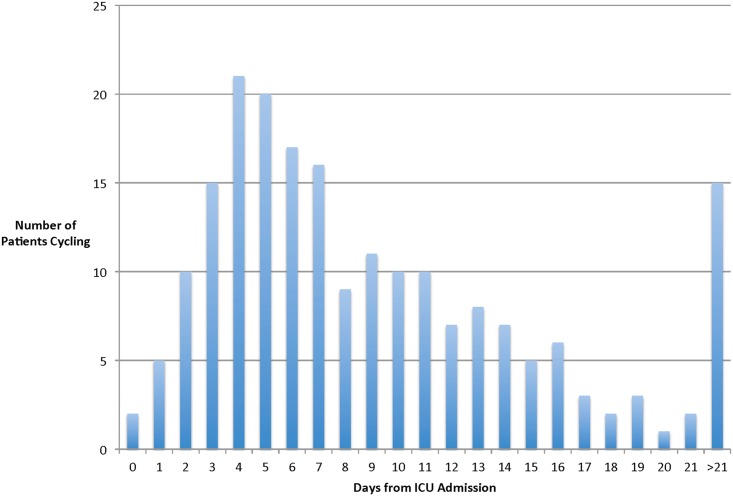
Histogram of cycling by day of ICU stay. This figure outlines the number of patients biking by days since ICU admission. Of 205 in bed cycling sessions, over half (106 (52%)) occurred within the first 7 days of the patient’s ICU admission.

Of 205 cycling sessions, 150 (73.1%) occurred while patients received MV (via oral ETT (n = 144, 96.0%) or via tracheostomy (n = 6, 4.0%)), and the mean (SD) FiO_2_ was 38%(15). Over half (106 (51.7%) of all sessions occurred within the first 7 days of patients’ admission to the ICU (Fig 3). The mean (SD) MODS score [25] on cycling days was 3.4(2.9). Any infusions of benzodiazepines, opiates, propofol, or any bolus of benzodiazepine, opiate or propofol occurred during 40 (19.5%), 44 (21.5%), 38 (18.5%), and 77 (37.6%) of all sessions, respectively. The mean (SD) RASS score was -1.4 (1.6). Patients received vasopressors or inotropes during 6 (2.9%), and dialysis during 4 (2.0%) of all sessions (continuous renal replacement therapy, n = 3; hemodialysis, n = 1).

The median [IQR] distance cycled per session and per patient was 1.0 [0.9, 2.2] and 8.7 [5.0, 14.0] km, respectively. The maximum distance cycled per session, and per patient were 9.0 and 41.2 km, respectively. Therapists observed active cycling in 165 (80.5%) of all sessions. On days of in-bed cycling, the 3 most common additional PT interventions were passive range of motion (39 days, 19.0%), bed mobility (32, 15.6%), and airway clearance techniques (28, 13.7%). Sitting at the edge of the bed, standing, and walking occurred on 27 (13.2%), 24 (11.7%), and 7 (3.4%) of in-bed cycling days, respectively. On 86 (42%) days of in-bed cycling, no additional PT interventions occurred. Additional information on non-cycling physiotherapy interventions and cycling details per patient are available in S4 and S5 Tables.

### Strength and Functional Outcomes

At ICU discharge, 7/28 (25.0%) of all survivors were walking, which improved to 18/23 (78.3%) by hospital discharge (Table 5). For 20 survivors with paired assessments, patients’ Katz ADL and FSS-ICU scores were significantly lower at hospital discharge than at baseline (Tables 2 and 5, p-value for difference, Katz = 0.004; FSS-ICU = 0.015). At hospital discharge, patients required assistance for at least 2 ADLs as well as for standing or walking.

**Table 5:** Patient strength and functional outcomes.

	ICU Awakening	ICU Discharge	Hospital Discharge
	N = 28^a^	N = 26^b^	N = 20^c^
**Muscle Strength**			
Medical Research Council (MRC) Sum Score	47.9 (9.4)	47.4 (12.9)	54.1 (5.3)
Total score <48, n (%)	10 (47.6)	10 (47.6)	4 (23.5)
Hand Grip Strength, median [IQR] kg	8.8 [3.0 to 16.5]	10.8 [3.8 to 18.3]	16.3 [10.2 to 21.5]
Knee Extensor Strength (N)	73.8 (79.1)	69.9 (72.7)	73.2 (85.4)
**Function**			
Katz ADL score	0.32 (0.94)	0.73 (1.48)	3.85 (2.30)
Functional Status Score for ICU	15.0 (8.9)	19.2 (10.7)	28.7 (8.2)
Physical Function Test for ICU-scored	4.6 (1.7)	5.3 (2.1)	7.2 (1.3)
6 Minute Walk Test (metres)	-	114 (-)	343 (-)

This table outlines the strength and function outcomes recorded at ICU awakening, ICU discharge, and hospital discharge. All values are mean (SD) unless otherwise specified. Abbreviations: IQR = interquartile range; ADL = activities of daily living; ICU = intensive care unit.

^a^Sample size for assessments performed at ICU Awakening: MRC Sum Score and MRC total score <48, 21; Hand grip, 22; Knee extensor strength, 14; Katz ADL score, 28; Functional Status Score for ICU, 23; Physical Function Test for ICU, 26; 6 minute walk test, 0.

^b^Sample size for assessments performed at ICU Discharge: MRC Sum Score and MRC total score <48, 21; Hand grip, 22; Knee extensor strength, 19; Katz ADL score, 26; Functional Status Score for ICU, 25; Physical Function Test for ICU, 26; 6 minute walk test, 1.

^c^Sample size for assessments performed at Hospital Discharge: MRC Sum Score, MRC total score <48, and Hand grip, 17; Knee extensor strength, 14; Katz ADL score, 20; Functional Status Score for ICU and Physical Function Test for ICU, 20; 6 minute walk test, 1.

## Discussion

Our results suggest that it is safe and feasible to enroll critically ill, hemodynamically stable MV patients in a rehabilitation study of early cycling. In this study of 33 MV patients, we began cycling within 3 days of ICU admission, session termination was infrequent, and device dislodgements did not occur. On average, patients received 5 cycling sessions of 30 minutes duration, cycled 1 km per session, and cycled a distance of 9 km in total in the ICU.

Our data add to a growing body of literature suggesting early cycling can occur safely with critically ill patients. A single, 20-minute passive cycling session started within the first 72 hours of MV documented no safety concerns while patients received low-dose vasoactive drug infusions, and no increase in cardiac output, or oxygen consumption.[13]. In a retrospective study of cycling incorporated into routine PT interventions in a medical ICU, cycling began within 4 days of MICU admission, and only 1 device dislodgement occurred out of 541 sessions (0.2% event rate).[17] In a case-control study of cycling and functional electrical stimulation initiated within the first 96 hours of MV, 1 transient desaturation occurred in 69 sessions.[13,16]

Due to the dynamic nature of critical illness, patients’ suitability for rehabilitation may vary daily. We designed our study to start cycling within the first 4 days of MV. Of 179 potentially eligible patients, 114 met some (or multiple) exclusion criteria. Of 144 exclusion reasons, almost half (68, 47%) occurred because patients had one or more persistent temporary exemptions precluding cycling within the first 4 days of MV (Fig 2). Most temporary exemptions reflected patients’ acuity: receipt of neuromuscular blocking agents (n = 19), increasing vasoactive medications (n = 14), or active myocardial ischemia/uncontrolled arrhythmia (n = 8). During our study, new studies supporting the safety of rehabilitation (including cycling) with femoral catheters were published[37,38]; we subsequently revised our protocol to remove this exemption. Future studies need to consider the possible impact of temporary exemptions from cycling in ICU patients and their impact on patient outcomes, and document protocol adherence, as in other critical care trials.

Prospective research in early cycling is feasible. We attained a high consent rate (92%), met our target sample size, delivered early cycling, and measured physical function in all survivors at hospital discharge. Previous early rehabilitation studies [9,11,14,39–41] had consent rates varying from 48% [42] to 89% [11], indicating differing receptivity of substitute decision makers to early rehabilitation research. We met our recruitment target, whereas some studies closed early due to slow accrual.[14,40,41] While another early rehabilitation intervention, NMES, had difficulty consistently achieving muscle contractions[40,43], all but 1 patient in this study successfully received cycling. However, of 31 patients eligible not enrolled, 22 (71%) were not enrolled because physiotherapists did not have capacity to manage multiple cycling trial patients concurrently (Fig 2). Investigators need to consider how to best engage front line ICU staff in delivering and/or outcome measure assessment to optimize timely accrual and cycling opportunities.

Further prospective research on the efficacy of early cycling in medical-surgical MV patients is needed. Cycling targets the legs, particularly hip flexors, which are most vulnerable to muscle atrophy and weakness during bed rest [44]. Two RCTs of cycling as part of a multimodal intervention showed no functional differences between intervention and control groups [14,15]. In these studies, cycling occurred later in the patients’ rehabilitation, and it was difficult to discern the unique contribution of cycling to patient outcomes[14,15]. In a retrospective cohort of cycling in a medical ICU, patients received 25 minutes of cycling in 2 of 4 total PT sessions during their stay, but functional status was not reported[17]. Cycling may offer a rehabilitation option for a broad range of ICU patients, particularly those who must be bedbound, have ~75° knee and ~80° available hip flexion[17], are not on active spinal precautions, and have no other orthopedic restrictions (e.g., no weight-bearing). However, further research in this area is needed.

Early mobility is recommended as a front-line non-pharmacological intervention to reduce the incidence and duration of delirium in critically ill patients.[45] However, some mobilization protocols require patients to be interactive [41,46], which may delay the time to start rehabilitation during the early critical time period for muscle size and strength losses. A multicenter prospective cohort study reported the presence of an oral ETT as one of the main barriers to mobilization in 192 MV patients.[10] Only 37% of all patients received any mobilization within the first 14 days of MV, and only in 16% of all potential occasions.[10] In contrast, patients in our cohort started cycling within a median of 3 days of MV, over half of all sessions occurred within the first 7 days of ICU admission (Fig 3), and 70% occurred while patients received MV via ETT.

### Limitations and Strengths

We had no control group to determine if cycling improved patient outcomes compared to usual care. Experienced ICU PTs conducted all biking sessions and outcome measures. Compared to other ICU cycling studies,[13,47] few patients cycled while receiving vasopressors or inotropes in our cohort, reflecting the predominant respiratory conditions in our population. We did not systematically record patients’ delirium status or reasons why they refused cycling. We conducted this study in a single centre ICU with a highly collaborative PT department and strong interprofessional critical care research culture.

To our knowledge, this is the largest prospective cohort of early cycling sessions in MV, medical-surgical ICU patients. We included an a-priori sample size calculation focused on safety events, prospectively collected all data, and engaged clinical PTs to lead all cycling sessions. We had a high consent rate, achieved our sample size target, and patients received multiple cycling sessions. Our Safety (termination) event rate of 2% was similar to the RCT of cycling started 2 weeks after ICU admission, where 3.8% (16/425) of sessions terminated early.[11] While we originally excluded patients from cycling if they had femoral catheters in-situ, we revised our protocol during the study to reflect new evidence supporting the safety of mobility activities with femoral catheters.[37,38]

## Conclusions

This study suggests that it is safe and feasible for hemodynamically stable MV patients to receive early cycling in the ICU and may inform future RCTs in this field.

## Supporting Information

S1 Fig TryCYCLE Study Schema. This diagram outlines the TryCYCLE study schema. Abbreviations: MV = mechanical ventilation; ICU = intensive care unit; PT = physiotherapy interventions.
(DOCX)

S1 Table Transparent Reporting of Evaluations with Nonrandomized Designs (TREND) Statement Checklist. (DOCX)

S2 Table Template for Intervention Description and Replication (TIDieR) Checklist. (DOCX)

S3 Table Characteristics of physiologic changes during in-bed cycling sessions. Values in this table represent vital sign recordings during in-bed cycling sessions. All values represent mean (SD). * = p<0.001 difference between pre- and post- cycling heart rate; ** p = 0.004 difference between pre- and post- cycling mean arterial pressure. ^a^Sample size for blood pressure measurements at 5, 10, 20, and 30 minutes: n = 201, n = 190, n = 178, and n = 146, respectively. Abbreviations: bpm = beats per minute.
(DOCX)

S4 Table Physiotherapy interventions occurring on 205 days of in-bed cycling. In this table, we outline additional therapeutic activities occurring on days of in-bed cycling.
(DOCX)

S5 Table Individual patient in-bed cycling details. This table outlines the mean (SD) number of cycling sessions, cycling session duration, and distance per cycling session for all patients. Abbreviations: SD = standard deviation.
(DOCX)

S1 File Study Protocol Approved by Hamilton Integrated Research Ethics Board. (PDF)

S1 Text Canadian Critical Care Trials Group Membership as of November 2016. (DOCX)
