# Supplementary material for: TryCYCLE: A Prospective Study of the Safety and Feasibility of Early In-Bed Cycling in Mechanically Ventilated Patients
Source: PLoS One. 2016 Dec 28;11(12):e0167561. doi: 10.1371/journal.pone.0167561 (PMC5193383; doi:10.1371/journal.pone.0167561)
Supplement: S1 Fig — This diagram outlines the TryCYCLE study schema. Abbreviations: MV = mechanical ventilation; ICU = intensive care unit; PT = physiotherapy interventions. (DOCX) [file pone.0167561.s001.docx]

**TryCYCLE: A prospective study of the safety and feasibility of early in-bed cycling in mechanically ventilated patients**

^1,2,3^Michelle E Kho, PT, PhD, ^2^Alexander J Molloy, BSc, ^4^France Clarke, RRT, ^2^Daana Ajami, MSc(PT), ^2^Magda McCaughan, MSc(PT), ^2^Kristy Obrovac, MSc(PT), ^2^Christina Murphy, MSc(PT), ^2^Laura Camposilvan, BSc, ^5^Margaret S Herridge, MD, MPH, ^6,7^Karen KY Koo, MD, FRCPC, MSc, ^8^Jill Rudkowski MD, FRCPC, ^9^Andrew JE Seely MD, PhD, FRCSC, ^3^Jennifer M Zanni, PT, DScPT, CCS, ^10^Marina Mourtzakis, PhD, ^11^Thomas Piraino, RRT, ^3,8^Deborah J Cook, MD, FRCPC, MSc and the Canadian Critical Care Trials Group

**Supporting Information:**

**S1 Fig. TryCYCLE Study Schema.** This diagram outlines the TryCYCLE study schema. Abbreviations: MV= mechanical ventilation; ICU = intensive care unit; PT = physiotherapy interventions.

**S1 Table. Transparent Reporting of Evaluations with Nonrandomized Designs (TREND) Statement Checklist.**

**S2 Table.** **Template for Intervention Description and Replication (TIDieR) Checklist.**

**S3 Table.** **Characteristics of physiologic changes during in-bed cycling sessions.** Values in this table represent vital sign recordings during in-bed cycling sessions. All values represent mean (SD). *= p<0.001 difference between pre- and post- cycling heart rate; ** p=0.004 difference between pre- and post- cycling mean arterial pressure. ^a^Sample size for blood pressure measurements at 5, 10, 20, and 30 minutes: n=201, n=190, n=178, and n=146, respectively. Abbreviations: bpm = beats per minute.

**S4 Table.** **Physiotherapy interventions occurring on 205 days of in-bed cycling.** In this table, we outline additional therapeutic activities occurring on days of in-bed cycling.

**S5 Table.** **Individual patient in-bed cycling details.** This table outlines the mean (SD) number of cycling sessions, cycling session duration, and distance per cycling session for all patients. Abbreviations: SD = standard deviation.

**Supplemental Figure 1**: TryCYCLE Study Schema


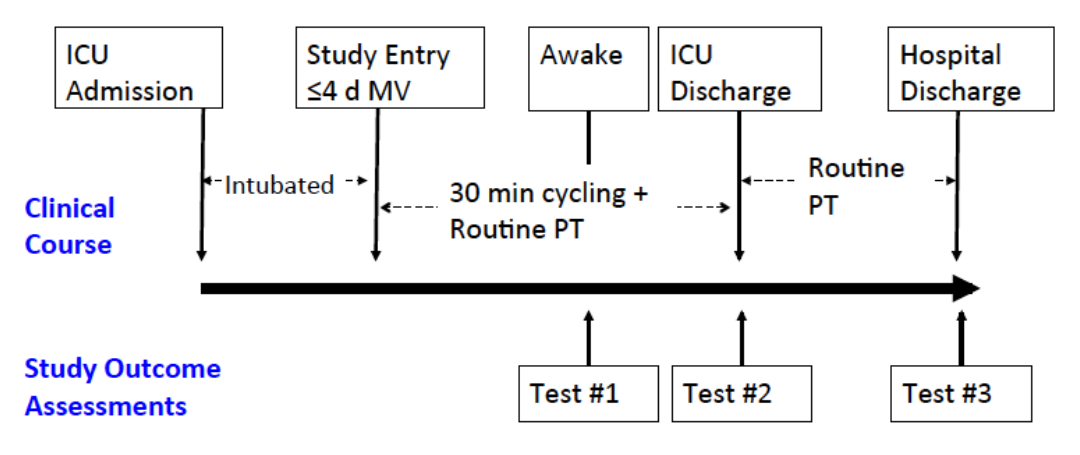


Legend: This diagram outlines the TryCYCLE study schema. Abbreviations: MV= mechanical ventilation; ICU = intensive care unit; PT = physiotherapy interventions

**Supplemental Table 1**: Transparent Reporting of Evaluations with Nonrandomized Designs (TREND) Statement Checklist

| Paper Section/ Topic | Item No | Descriptor | Reported? | | |  |  |
| --- | --- | --- | --- | --- | --- | --- | --- |
|  |  |  | ✔ | | Pg # |  |  |
| Title and Abstract | | | | | |  |  |
| Title and Abstract | 1 | Information on how unit were allocated to interventions | ✔ | | 1 |  |  |
|  |  | Structured abstract recommended | ✔ | | 3 |  |  |
|  |  | Information on target population or study sample | ✔ | | 3 |  |  |
| Introduction | | | | | |  |  |
| Background | 2 | Scientific background and explanation of rationale | ✔ | | 4-5 |  |  |
|  |  | Theories used in designing behavioral interventions | Not applicable | | |  |  |
| Methods | | | | | |  |  |
| Participants | 3 | Eligibility criteria for participants, including criteria at different levels in recruitment/sampling plan (e.g., cities, clinics, subjects) | ✔ | | Table 1 |  |  |
|  |  | Method of recruitment (e.g., referral, self-selection), including the sampling method if a systematic sampling plan was implemented | RC screen | | 5 |  |  |
|  |  | Recruitment setting | ICU | | 5 |  |  |
|  |  | Settings and locations where the data were collected | ✔ | | 5 |  |  |
| Interventions | 4 | Details of the interventions intended for each study condition and how and when they were actually administered, specifically including: |  | |  |  |  |
|  |  | - Content: what was given? | cycling | | 7 |  |  |
|  |  | - Delivery method: how was the content given? | In-person | | 7 |  |  |
|  |  | - Unit of delivery: how were the subjects grouped during delivery? | Per person | | 7 |  |  |
|  |  | - Deliverer: who delivered the intervention? | PTs | | 7 |  |  |
|  |  | - Setting: where was the intervention delivered? | ICU | | 7 |  |  |
|  |  | - Exposure quantity and duration: how many sessions or episodes or events were intended to be delivered? How long were they intended to last? | 6 d/ wk to ICU d/c or 28 days | | 7 |  |  |
|  |  | - Time span: how long was it intended to take to deliver the intervention to each unit? | 31 minutes | | 7 |  |  |
|  |  | - Activities to increase compliance or adherence (e.g., incentives) | Not applicable | | |  |  |
| Objectives | 5 | Specific objectives and hypotheses | ✔ | 5 | |  |  |
| Outcomes | 6 | Clearly defined primary and secondary outcome measures | ✔ | 8 | |  |  |
|  |  | Methods used to collect data and any methods used to enhance the quality of measurements | ✔ | 7, 8 | |  |  |
|  |  | Information on validated instruments such as psychometric and biometric properties | ✔ | 8 | |  |  |
| Sample Size | 7 | How sample size was determined and, when applicable, explanation of any interim analyses and stopping rules | ✔ | 8 | |  |  |
| Assignment Method | 8 | Unit of assignment (the unit being assigned to study condition, e.g., individual, group, community) | ✔ | 5 | |  |  |
|  |  | Method used to assign units to study conditions, including details of any restriction (e.g., blocking, stratification, minimization) | Not applicable | | |  |  |
|  |  | Inclusion of aspects employed to help minimize potential bias induced due to non-randomization (e.g., matching) | Not applicable | | |  |  |
| Blinding (masking) | 9 | Whether or not participants, those administering the interventions, and those assessing the outcomes were blinded to study condition assignment; if so, statement regarding how the blinding was accomplished and how it was assessed. | Open-label | 5 | |  |  |
| Unit of Analysis | 10 | Description of the smallest unit that is being analyzed to assess intervention effects (e.g., individual, group, or community) | Per patient | 9 | |  |  |
|  |  | If the unit of analysis differs from the unit of assignment, the analytical method used to account for this (e.g., adjusting the standard error estimates by the design effect or using multilevel analysis) | Not applicable | | |  |  |
| Statistical Methods | 11 | Statistical methods used to compare study groups for primary methods outcome(s), including complex methods of correlated data | ✔ | 9 | |  |  |
|  |  | Statistical methods used for additional analyses, such as a subgroup analyses and adjusted analysis | ✔ | 9 | |  |  |
|  |  | Methods for imputing missing data, if used | Not applicable | | |  |  |
|  |  | Statistical software or programs used |  | 9 | |  |  |
| Results | | | |  | |  | |
| Participant flow | 12 | Flow of participants through each stage of the study: enrollment, assignment, allocation, and intervention exposure, follow-up, analysis (a diagram is strongly recommended) | ✔ | Figure 2 | |  |  |
|  |  | - Enrollment: the numbers of participants screened for eligibility, found to be eligible or not eligible, declined to be enrolled, and enrolled in the study | ✔ | Figure 2 | |  |  |
|  |  | - Assignment: the numbers of participants assigned to a study condition | ✔ | 9 | |  |  |
|  |  | - Allocation and intervention exposure: the number of participants assigned to each study condition and the number of participants who received each intervention | ✔ | 9 | |  |  |
|  |  | - Follow-up: the number of participants who completed the follow- up or did not complete the follow-up (i.e., lost to follow-up), by study condition | ✔ | Figure 2 | |  |  |
|  |  | - Analysis: the number of participants included in or excluded from the main analysis, by study condition | ✔ | 9 | |  |  |
|  |  | - Description of protocol deviations from study as planned, along with reasons | ✔ | Table 4 | |  |  |
| Recruitment | 13 | Dates defining the periods of recruitment and follow-up | ✔ | 9 | |  |  |
| Baseline Data | 14 | Baseline demographic and clinical characteristics of participants in each study condition | ✔ | Table 2 | |  |  |
|  |  | Baseline characteristics for each study condition relevant to specific disease prevention research | Not applicable | | | |  |
|  |  | Baseline comparisons of those lost to follow-up and those retained, overall and by study condition | Not applicable | | | |  |
|  |  | Comparison between study population at baseline and target population of interest | Not applicable | | | |  |
| Baseline equivalence | 15 | Data on study group equivalence at baseline and statistical methods used to control for baseline differences | Not applicable | | | |  |
| Numbers analyzed | 16 | Number of participants (denominator) included in each analysis for each study condition, particularly when the denominators change for different outcomes; statement of the results in absolute numbers when feasible | ✔ | | S2-4 Tables; 9-16 | |  |
|  |  | Indication of whether the analysis strategy was “intention to treat” or, if not, description of how non-compliers were treated in the analyses | ✔ | | 9-16 | |  |
| Outcomes and estimation | 17 | For each primary and secondary outcome, a summary of results for each estimation study condition, and the estimated effect size and a confidence interval to indicate the precision | ✔ | | 9-16 | |  |
|  |  | Inclusion of null and negative findings | Not applicable | | | |  |
|  |  | Inclusion of results from testing pre-specified causal pathways through which the intervention was intended to operate, if any | Not applicable | | | |  |
| Ancillary analyses | 18 | Summary of other analyses performed, including subgroup or restricted analyses, indicating which are pre-specified or exploratory | Not applicable | | | |  |
| Adverse events | 19 | Summary of all important adverse events or unintended effects in each study condition (including summary measures, effect size estimates, and confidence intervals) | ✔ | | 11, Table 3 | |  |
| DISCUSSION | | |  | |  | |  |
| Interpretation | 20 | Interpretation of the results, taking into account study hypotheses, sources of potential bias, imprecision of measures, multiplicative analyses, and other limitations or weaknesses of the study | ✔ | | 16-19 | |  |
|  |  | Discussion of results taking into account the mechanism by which the intervention was intended to work (causal pathways) or alternative mechanisms or explanations | Not applicable, feasibility study | | | |  |
|  |  | Discussion of the success of and barriers to implementing the intervention, fidelity of implementation | ✔ | | 16-19 | |  |
|  |  | Discussion of research, programmatic, or policy implications | ✔ | | 16-19 | |  |
| Generalizability | 21 | Generalizability (external validity) of the trial findings, taking into account the study population, the characteristics of the intervention, length of follow-up, incentives, compliance rates, specific sites/settings involved in the study, and other contextual issues | ✔ | | 16-19 | |  |
| Overall Evidence | 22 | General interpretation of the results in the context of current evidence and current theory | ✔ | | 16-19 | |  |

*From:* Des Jarlais, D. C., Lyles, C., Crepaz, N., & the Trend Group (2004). Improving the reporting quality of nonrandomized evaluations of behavioral and public health interventions: The TREND statement. *American Journal of Public Health*, 94, 361-366.

**Supplemental Table 2**: Template for Intervention Description and Replication (TIDieR) Checklist

**
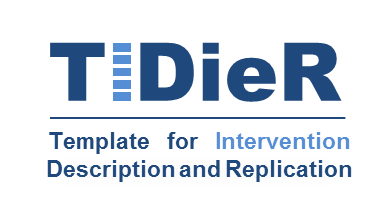
The TIDieR (Template for Intervention Description and Replication) Checklist*:**

Information to include when describing an intervention and the location of the information

| **Item number** | **Item** | **Where located **** | |
| --- | --- | --- | --- |
|  |  | Primary paper  (page or appendix  number) | Other ^†^ (details) |
|  | **BRIEF NAME** |  |  |
| **1.** | Provide the name or a phrase that describes the intervention. | _____1_______ | ______________ |
|  | **WHY** |  |  |
| **2.** | Describe any rationale, theory, or goal of the elements essential to the intervention. | _____4-5_____ | _____________ |
|  | **WHAT** |  |  |
| **3.** | Materials: Describe any physical or informational materials used in the intervention, including those provided to participants or used in intervention delivery or in training of intervention providers. Provide information on where the materials can be accessed (e.g. online appendix, URL). | _____5-8_____ | _____________ |
| **4.** | Procedures: Describe each of the procedures, activities, and/or processes used in the intervention, including any enabling or support activities. | _____5-8_____ | _____________ |
|  | **WHO PROVIDED** |  |  |
| **5.** | For each category of intervention provider (e.g. psychologist, nursing assistant), describe their expertise, background and any specific training given. | _____7-8______ | _____________ |
|  | **HOW** |  |  |
| **6.** | Describe the modes of delivery (e.g. face-to-face or by some other mechanism, such as internet or telephone) of the intervention and whether it was provided individually or in a group. | _____7______ | _____________ |
|  | **WHERE** |  |  |
| **7.** | Describe the type(s) of location(s) where the intervention occurred, including any necessary infrastructure or relevant features. | _____5-7______ | _____________ |
|  | **WHEN and HOW MUCH** |  |  |
| **8.** | Describe the number of times the intervention was delivered and over what period of time including the number of sessions, their schedule, and their duration, intensity or dose. | ______7_____ | _____________ |
|  | **TAILORING** |  |  |
| **9.** | If the intervention was planned to be personalised, titrated or adapted, then describe what, why, when, and how. | ______7______ | _____________ |
|  | **MODIFICATIONS** |  |  |
| **10.^ǂ^** | If the intervention was modified during the course of the study, describe the changes (what, why, when, and how). | Table 2 | __Femoral catheters removed as exemption |
|  | **HOW WELL** |  |  |
| **11.** | Planned: If intervention adherence or fidelity was assessed, describe how and by whom, and if any strategies were used to maintain or improve fidelity, describe them. | ____7____ | _________ |
| **12.^ǂ^** | Actual: If intervention adherence or fidelity was assessed, describe the extent to which the intervention was delivered as planned. | Table 4 | _________ |

*From***:** Hoffmann TC, Glasziou PP, Boutron I, Milne R, Perera R, Moher D, Altman DG, Barbour V, Macdonald H, Johnston M, Lamb SE, Dixon-Woods M, McCulloch P, Wyatt JC, Chan AW, Michie S. Better reporting of interventions: template for intervention description and replication (TIDieR) checklist and guide. BMJ. 2014 Mar 7;348:g1687.

**Supplemental Table 3:** Characteristics of physiologic changes during in-bed cycling sessions

| **Measurement** | **Pre-cycling**  **N=205** | **5 min**  **N=202** | **10 min**  **N=191** | **20 min**  **N=179** | **30 min**  **N=148** | **Post-cycling**  **N=205** |
| --- | --- | --- | --- | --- | --- | --- |
| Heart rate (bpm) | 85.3 (15.4) | 86.4 (15.5) | 86.1 (15.4) | 85.6 (15.7) | 84.6 (14.7) | 87.5 (15.9)* |
| Systolic blood pressure (mmHg)^a^ | 135.1 (19.2) | 138.2 (20.3) | 137.1 (20.1) | 135.8 (21.5) | 135.2 (20.2) | 136.2 (20.3) |
| Diastolic blood pressure (mmHg)^a^ | 65.6 (10.4) | 67.3 (10.5) | 67.1 (11.2) | 65.8 (10.7) | 65.2 (11.5) | 66.6 (12.2) |
| Mean arterial pressure (mmHg)^a^ | 87.4 (12.0) | 90.1 (12.6) | 89.6 (12.6) | 88.6 (12.4) | 88.0 (12.9) | 89.1 (13.0)** |
| Percutaneous SpO_2_ | 95.6 (2.7) | 95.3 (2.9) | 95.3 (2.8) | 95.3 (2.9) | 95.3 (3.2) | 95.3 (3.2) |
| FiO_2_ | 37.8 (14.7) | 38.4 (15.7) | 38.8 (15.8) | 38.3 (15.0) | 37.3 (13.8) | 38.7 (15.8) |

**Legend**: Values in this table represent vital sign recordings during in-bed cycling sessions. All values represent mean (SD). *= p<0.001 difference between pre- and post- cycling heart rate; ** p=0.004 difference between pre- and post- cycling mean arterial pressure. ^a^Sample size for blood pressure measurements at 5, 10, 20, and 30 minutes: n=201, n=190, n=178, and n=146, respectively. Abbreviations: bpm = beats per minute.

**Supplemental Table 4**: Physiotherapy interventions occurring on 205 days of in-bed cycling

| **Non-cycling physiotherapy interventions** | **N (%)** |
| --- | --- |
| Passive range of motion | 39 (19.0) |
| Bed mobility | 32 (15.6) |
| Chest physiotherapy/airway clearance | 28 (13.7) |
| Dangle | 27 (13.2) |
| Active range of motion | 26 (12.7) |
| Standing | 24 (11.7) |
| Active assisted range of motion | 18 (8.8) |
| Active transfer from bed to chair | 11 (5.4) |
| Walking | 7 (3.4) |
| Patient refused additional activities | 2 (1.7) |
| No additional activities reported | 86 (42.0) |

Legend: In this table, we outline additional therapeutic activities occurring on days of in-bed cycling.

**Supplemental Table 5**: Individual patient in-bed cycling details

| **Patient #** | **Number of Sessions** | **Duration in Minutes** | **Distance in km** |
| --- | --- | --- | --- |
|  |  | **Mean (SD)** | **Mean (SD)** |
| 1 | 2 | 30.7 (0.6) | 4.9 (1.1) |
| 2 | 2 | 24.0 (10.1) | 2.6 (0.1) |
| 3 | 7 | 31.5 (0.7) | 2.6 (0.3) |
| 4 | 6 | 22.2 (5.8) | 2.3 (0.8) |
| 5 | 1 | 31.6 (-) | 3.5 (-) |
| 6 | 5 | 30.8 (0.9) | 1.5 (0.8) |
| 7 | 5 | 16.1 (13.9) | 1.6 (2.0) |
| 8 | 12 | 30.8 (0.5) | 0.9 (0.04) |
| 9 | 8 | 21.4 (10.3) | 1.4 (1.7) |
| 10 | 2 | 21.3 (13.3) | 3.7 (2.9) |
| 11 | 6 | 30.7 (0.08) | 6.9 (0.3) |
| 12 | 4 | 25.9 (10.1) | 0.9 (0.05) |
| 13 | 12 | 29.1 (5.6) | 1.1 (0.3) |
| 14 | 8 | 30.7 (0.04) | 3.8 (2.8) |
| 15 | 4 | 26.2 (9.4) | 1.8 (1.6) |
| 16 | 13 | 18.4 (12.9) | 0.6 (0.4) |
| 17 | 2 | 30.7 (0.04) | 0.9 (0.01) |
| 18 | 4 | 30.7 (0.05) | 1.1 (0.2) |
| 19 | 2 | 21.4 (13.0) | 2.5 (2.0) |
| 21 | 3 | 30.6 (0.01) | 7.9 (1.0) |
| 22 | 15 | 20.4 (10.7) | 0.7 (0.4) |
| 23 | 8 | 21.4 (7.0) | 1.9 (1.1) |
| 24 | 4 | 20.9 (2.9) | 4.0 (1.7) |
| 25 | 8 | 23.3 (9.1) | 2.6 (1.9) |
| 26 | 6 | 24.1 (10.1) | 1.5 (1.0) |
| 27 | 2 | 21.9 (5.1) | 2.2 (1.0) |
| 28 | 5 | 25.5 (10.5) | 1.1 (0.4) |
| 29 | 14 | 16.6 (8.2) | 1.6 (0.9) |
| 30 | 12 | 29.5 (3.0) | 0.9 (0.2) |
| 31 | 5 | 30.5 (0.3) | 0.9 (0.02) |
| 32 | 14 | 27.9 (6.6) | 0.9 (0.2) |
| 33 | 3 | 27.0 (5.4) | 0.8 (0.2) |
| 34 | 1 | 21.0 (-) | 0.8 (-) |

Legend: This table outlines the mean (SD) number of cycling sessions, cycling session duration, and distance per cycling session for all patients. Abbreviations: SD = standard deviation.
