# Supplementary material for: TryCYCLE: A Prospective Study of the Safety and Feasibility of Early In-Bed Cycling in Mechanically Ventilated Patients
Source: PLoS One. 2016 Dec 28;11(12):e0167561. doi: 10.1371/journal.pone.0167561 (PMC5193383; doi:10.1371/journal.pone.0167561)
Supplement: S3 Table — Values in this table represent vital sign recordings during in-bed cycling sessions. All values represent mean (SD). * = p<0.001 difference between pre- and post- cycling heart rate; ** p = 0.004 difference between pre- and post- cycling mean arterial pressure. aSample size for blood pressure measurements at 5, 10, 20, and 30 minutes: n = 201, n = 190, n = 178, and n = 146, respectively. Abbreviations: bpm = beats per minute. (DOCX) [file pone.0167561.s004.docx]

**Supplemental Table 3:** Characteristics of physiologic changes during in-bed cycling sessions

| **Measurement** | **Pre-cycling**  **N=205** | **5 min**  **N=202** | **10 min**  **N=191** | **20 min**  **N=179** | **30 min**  **N=148** | **Post-cycling**  **N=205** |
| --- | --- | --- | --- | --- | --- | --- |
| Heart rate (bpm) | 85.3 (15.4) | 86.4 (15.5) | 86.1 (15.4) | 85.6 (15.7) | 84.6 (14.7) | 87.5 (15.9)* |
| Systolic blood pressure (mmHg)^a^ | 135.1 (19.2) | 138.2 (20.3) | 137.1 (20.1) | 135.8 (21.5) | 135.2 (20.2) | 136.2 (20.3) |
| Diastolic blood pressure (mmHg)^a^ | 65.6 (10.4) | 67.3 (10.5) | 67.1 (11.2) | 65.8 (10.7) | 65.2 (11.5) | 66.6 (12.2) |
| Mean arterial pressure (mmHg)^a^ | 87.4 (12.0) | 90.1 (12.6) | 89.6 (12.6) | 88.6 (12.4) | 88.0 (12.9) | 89.1 (13.0)** |
| Percutaneous SpO_2_ | 95.6 (2.7) | 95.3 (2.9) | 95.3 (2.8) | 95.3 (2.9) | 95.3 (3.2) | 95.3 (3.2) |
| FiO_2_ | 37.8 (14.7) | 38.4 (15.7) | 38.8 (15.8) | 38.3 (15.0) | 37.3 (13.8) | 38.7 (15.8) |

**Legend**: Values in this table represent vital sign recordings during in-bed cycling sessions. All values represent mean (SD). *= p<0.001 difference between pre- and post- cycling heart rate; ** p=0.004 difference between pre- and post- cycling mean arterial pressure. ^a^Sample size for blood pressure measurements at 5, 10, 20, and 30 minutes: n=201, n=190, n=178, and n=146, respectively. Abbreviations: bpm = beats per minute.
