# Supplementary material for: TryCYCLE: A Prospective Study of the Safety and Feasibility of Early In-Bed Cycling in Mechanically Ventilated Patients
Source: PLoS One. 2016 Dec 28;11(12):e0167561. doi: 10.1371/journal.pone.0167561 (PMC5193383; doi:10.1371/journal.pone.0167561)
Supplement: S4 Table — In this table, we outline additional therapeutic activities occurring on days of in-bed cycling. (DOCX) [file pone.0167561.s005.docx]

**Supplemental Table 4**: Physiotherapy interventions occurring on 205 days of in-bed cycling

| **Non-cycling physiotherapy interventions** | **N (%)** |
| --- | --- |
| Passive range of motion | 39 (19.0) |
| Bed mobility | 32 (15.6) |
| Chest physiotherapy/airway clearance | 28 (13.7) |
| Dangle | 27 (13.2) |
| Active range of motion | 26 (12.7) |
| Standing | 24 (11.7) |
| Active assisted range of motion | 18 (8.8) |
| Active transfer from bed to chair | 11 (5.4) |
| Walking | 7 (3.4) |
| Patient refused additional activities | 2 (1.7) |
| No additional activities reported | 86 (42.0) |

Legend: In this table, we outline additional therapeutic activities occurring on days of in-bed cycling.
