# Supplementary material for: TryCYCLE: A Prospective Study of the Safety and Feasibility of Early In-Bed Cycling in Mechanically Ventilated Patients
Source: PLoS One. 2016 Dec 28;11(12):e0167561. doi: 10.1371/journal.pone.0167561 (PMC5193383; doi:10.1371/journal.pone.0167561)
Supplement: S5 Table — This table outlines the mean (SD) number of cycling sessions, cycling session duration, and distance per cycling session for all patients. Abbreviations: SD = standard deviation. (DOCX) [file pone.0167561.s006.docx]

**Supplemental Table 5**: Individual patient in-bed cycling details

| **Patient #** | **Number of Sessions** | **Duration in Minutes** | **Distance in km** |
| --- | --- | --- | --- |
|  |  | **Mean (SD)** | **Mean (SD)** |
| 1 | 2 | 30.7 (0.6) | 4.9 (1.1) |
| 2 | 2 | 24.0 (10.1) | 2.6 (0.1) |
| 3 | 7 | 31.5 (0.7) | 2.6 (0.3) |
| 4 | 6 | 22.2 (5.8) | 2.3 (0.8) |
| 5 | 1 | 31.6 (-) | 3.5 (-) |
| 6 | 5 | 30.8 (0.9) | 1.5 (0.8) |
| 7 | 5 | 16.1 (13.9) | 1.6 (2.0) |
| 8 | 12 | 30.8 (0.5) | 0.9 (0.04) |
| 9 | 8 | 21.4 (10.3) | 1.4 (1.7) |
| 10 | 2 | 21.3 (13.3) | 3.7 (2.9) |
| 11 | 6 | 30.7 (0.08) | 6.9 (0.3) |
| 12 | 4 | 25.9 (10.1) | 0.9 (0.05) |
| 13 | 12 | 29.1 (5.6) | 1.1 (0.3) |
| 14 | 8 | 30.7 (0.04) | 3.8 (2.8) |
| 15 | 4 | 26.2 (9.4) | 1.8 (1.6) |
| 16 | 13 | 18.4 (12.9) | 0.6 (0.4) |
| 17 | 2 | 30.7 (0.04) | 0.9 (0.01) |
| 18 | 4 | 30.7 (0.05) | 1.1 (0.2) |
| 19 | 2 | 21.4 (13.0) | 2.5 (2.0) |
| 21 | 3 | 30.6 (0.01) | 7.9 (1.0) |
| 22 | 15 | 20.4 (10.7) | 0.7 (0.4) |
| 23 | 8 | 21.4 (7.0) | 1.9 (1.1) |
| 24 | 4 | 20.9 (2.9) | 4.0 (1.7) |
| 25 | 8 | 23.3 (9.1) | 2.6 (1.9) |
| 26 | 6 | 24.1 (10.1) | 1.5 (1.0) |
| 27 | 2 | 21.9 (5.1) | 2.2 (1.0) |
| 28 | 5 | 25.5 (10.5) | 1.1 (0.4) |
| 29 | 14 | 16.6 (8.2) | 1.6 (0.9) |
| 30 | 12 | 29.5 (3.0) | 0.9 (0.2) |
| 31 | 5 | 30.5 (0.3) | 0.9 (0.02) |
| 32 | 14 | 27.9 (6.6) | 0.9 (0.2) |
| 33 | 3 | 27.0 (5.4) | 0.8 (0.2) |
| 34 | 1 | 21.0 (-) | 0.8 (-) |

Legend: This table outlines the mean (SD) number of cycling sessions, cycling session duration, and distance per cycling session for all patients. Abbreviations: SD = standard deviation.
