# Supplementary material for: TryCYCLE: A Prospective Study of the Safety and Feasibility of Early In-Bed Cycling in Mechanically Ventilated Patients
Source: PLoS One. 2016 Dec 28;11(12):e0167561. doi: 10.1371/journal.pone.0167561 (PMC5193383; doi:10.1371/journal.pone.0167561)
Supplement: S1 File — (PDF) [file pone.0167561.s007.pdf]

"It is hard to convey just how debilitated one is after an insult of intensive care magnitude. When I was finally weaned (from mechanical ventilation), sitting in a chair was impossible….There was a remarkably persistent and overwhelming generalized weakness and fatigue."[1]

~Cheryl Misak, ICU Survivor

Survivors of the intensive care unit (ICU) face important physical function and mobility impairments, which can last up to 8 years post-ICU. With more patients surviving ICU due to advances in critical care, more are also at risk for post-ICU physical function impairments. Early rehabilitation, started in the ICU while a patient is receiving mechanical ventilation (MV), can improve patient outcomes and reduce ICU and hospital length of stay.

To date, only 2 randomized controlled trials (RCTs) of 192 patients have been conducted on early rehabilitation in the ICU. In a 2-centre, 104 patient RCT, 59% of those receiving early physiotherapy (PT) and occupational therapy within 1.5 days of MV returned to independent function at hospital discharge, compared to 35% of those receiving therapy within 7.4 days of MV ($p=0.02$). In a single-centre, 90 patient RCT, those receiving in-bed cycle ergometry and standard PT starting at ICU day 14 achieved 196 m in the 6-minute walk test at hospital discharge, compared to those receiving standard PT alone, who achieved 143 m ($p<0.05$). However, these studies have limitations. The early rehabilitation occurred in a medical ICU population, and not in a medical-surgical ICU population, which is more common in Canada. In-bed cycling, while studied in a medical-surgical population, did not start until the patients were 14 days into their ICU stay. **Thus, can we initiate in-bed cycle ergometry for patients early in their ICU stay, and will it improve patient outcomes?**

RESEARCH QUESTION: In medical-surgical ICU patients receiving MV, is it safe and feasible to initiate 30 minutes of in-bed cycling early after ICU admission and throughout the ICU stay?

TryCYCLE OBJECTIVES

1. Determine the **safety** of in-bed cycling initiated within 4 days of mechanical ventilation.
2. Determine the **feasibility** of conducting a future RCT of in-bed cycling in ICU.

OUTCOMES: We will consider our study **successful** if we achieve the following outcomes:

For Objective 1 (Safety):

a. **Adverse events**: The line or tube dislodgement rate is **low** (<4%).
b. **Cycling termination**: The *a priori* safety event rate is **low** (0 to 4%).
c. **Stability**: Patients **will tolerate in-bed cycling** while critically ill (heart and respiratory rates and blood pressure within 20% of baseline, no serious change in their variability).

For Objective 2 (Feasibility):

a. **Consent**: The consent rate is **high** (70%).
b. **Intervention delivery**: The daily research session delivery rate is **high** (>80%).
c. **Outcome measures**: Physical outcome measures are successfully performed in a **high** proportion of assessable patients at ICU awakening, ICU discharge, and hospital discharge (>80%).

SIGNIFICANCE: We have an urgent need to improve the rehabilitation and outcomes of an increasing number of ICU survivors. By 2026, as the baby boomer generation ages, the demand for ICU services in Canada is projected to increase by 40% compared to 2011. Thus, more Canadians will be at risk of disability following critical illness. Cycling is an attractive intervention because it can be applied to critically ill patients in bed, can be protocolized, administered by 1 clinician, and 1 trial to date suggests promising effects in long-stay ICU patients. Results from **Try-CYCLE** will inform the design and conduct of further research including a large-scale multicenter RCT evaluating the effectiveness of early in-bed cycling on patient-important outcomes.

RESEARCH PROPOSAL

1.0 OVERVIEW: IMPROVING PHYSICAL MOBILITY IN ICU SURVIVORS

Survivors of the intensive care unit (ICU) face important physical function and mobility impairments, which can last up to 8 years post-ICU.[2] With more patients surviving ICU due to medical advances, more are also at risk for post-ICU physical function impairments. Early rehabilitation, started in the ICU while a patient is receiving mechanical ventilation (MV), can improve patient outcomes, reduce ICU and hospital length of stay, and save inpatient hospitalization costs.[3-7]

To-date, only 2 randomized controlled trials (RCTs) of 192 patients form our best evidence for early rehabilitation in the ICU. In a 2-centre, 104 patient RCT, 59% of those receiving early physiotherapy (PT) and occupational therapy (OT) within 1.5 days of MV returned to independent function at hospital discharge, compared to 35% of those receiving therapy within 7.4 days of MV ($p=0.02$).[6] In a single-centre, 90 patient RCT, those receiving in-bed cycle ergometry starting at ICU day 14 and standard PT achieved 196 m in the 6-minute walk test at hospital discharge, compared to those receiving standard PT alone, who achieved 143 m ($p<0.05$).[3] However, these studies have limitations. The early rehabilitation occurred in a medical ICU population, and not in a medical-surgical ICU population, which is more common in Canada. In-bed cycling, while studied in a medical-surgical population, did not start until the patients were 14 days into their ICU stay. **To our knowledge, no one has systematically studied in-bed cycling started early in mechanically ventilated patients. Thus, is it safe and feasible to initiate a protocol of in-bed cycle ergometry for patients early in their ICU stay, and will it improve patient outcomes?**

RESEARCH QUESTION: In medical-surgical ICU patients receiving MV, is it safe and feasible to initiate 30 minutes of in-bed cycling early after ICU admission and throughout their ICU stay?

TryCYCLE OBJECTIVES

1. Determine the **safety** of in-bed cycling initiated within 4 days of mechanical ventilation.
2. Determine the **feasibility** of conducting a future RCT of in-bed cycling in ICU.

OUTCOMES: We will consider our study **successful** if we achieve the following outcomes:

For Objective 1 (Safety):

a. **Primary Outcome - Termination**: The *a priori* safety event rate is **low** (0 to 4%).
   Hypothesis: The termination event rate will not be different, or will be better (i.e., lower) than reported in the literature (0 to 4%).
b. **Adverse events**: The line or tube dislodgement rate is **low** (<4%).
   Hypothesis: The adverse event rate will not be different, or will be better (i.e., lower) than reported in the literature (<4%).
c. **Stability**: Patients will **tolerate in-bed cycling** while critically ill (heart and respiratory rates and blood pressure within 20% of baseline, no serious change in their variability).
   Hypothesis: Vital signs will not be different from pre-specified baseline levels (20% for HR, RR, and BP [systolic or diastolic]).

For Objective 2 (Feasibility):

a. **Consent**: The consent rate is **high** (70%).
   Hypothesis: The consent rate will not be different, or will be better (i.e., higher) than existing literature (70%).
b. **Intervention delivery**: The daily research session delivery rate is **high** (>80%).
   Hypothesis: The intervention delivery rate will not be different, or will be better (i.e., higher) than existing literature (>80%).

c. **Outcome measures**: Physical outcome measures are successfully performed in a **high** proportion of assessable patients at ICU awakening, ICU discharge, and hospital discharge (>80%).
   Hypothesis: The event rates for outcome measure ascertainment will be 80% or higher for each outcome measure, at each of the 3 time points (ICU awakening, ICU discharge, hospital discharge).

METHODS: This proposal is for a 33 patient, single-center, prospective observational cohort study in a 20-bed academic, tertiary medical-surgical ICU. We will recruit patients receiving MV for 24 hours or more, are within the first 4 days of MV, who could ambulate independently before ICU admission, and have no other exclusion criteria. Following consent, patients will receive 30 minutes of in-bed cycling 6 days per week, in addition to routine physiotherapy, for the duration of their ICU stay. We will determine the safety (**Objective 1**) and feasibility (**Objective 2**) of in-bed cycling in this population.

RESULTS / OUTCOMES: This is the first Canadian study in a comprehensive, multidisciplinary research program to inform a future multi-centre phase 3 RCT and economic evaluation of the effectiveness of early in-bed cycling on physical function and mobility in ICU survivors.

2.0 BACKGROUND AND RATIONALE

Critical care is the most costly hospital service. It accounts for 8.1% of inpatient hospital days, but represents 15.9% of inpatient direct expenses.[8] Between 1997-98 and 2003-04, over 2 million people received critical care in Canada (excluding Manitoba and Québec), representing 12.2% of hospital admissions.[8] While medical advances have reduced the mortality of respiratory failure,[9, 10] survival comes at a large and underappreciated human and societal cost.

Post-hospital discharge, ICU survivors incur important physical mobility impairments, and as a consequence, our Canadian healthcare system incurs substantial medical and rehabilitation costs. At 1 year follow-up, a landmark study documented how Canadian survivors of acute respiratory failure were 34% below age- and sex-matched norms for the 6-minute walk test (6MWT), which is a standardized measure of function and endurance, and 51% had not returned to work.[11] At 5-year follow-up these survivors remained 24% below 6MWT norms, and 23% still had not returned to work.[12]

Beyond hospital discharge, the healthcare utilization of ICU survivors is over 3 times greater than that of healthy workers.[12] Based on Canadian MV utilization projections for 2026,[13] a 75% survival rate, and a conservative estimate of 25% of survivors experiencing post-ICU disability in Ontario, **the potential cost of post-hospital healthcare utilization by ICU survivors in Canada is over $830 million at 5 years**. Moreover, the impact of critical illness in the elderly population may be more severe. Previously healthy elderly survivors of MV experienced, on average, 1.5 new mobility impairments compared to prospectively collected baseline functional status.[14] Elderly survivors of severe sepsis experienced important new cognitive and functional limits persisting for at least 8 years.[2]

By 2026, as the baby boomer generation ages, the demand for ICU services in Canada is projected to increase by 40% compared to 2011.[13] **We have an urgent need to proactively address the rehabilitation needs of ICU survivors.**

2.1 Is bed rest the right prescription for our critically ill patients?

Critically ill patients admitted to ICU are typically exposed to prolonged bed rest, which contributes to muscle atrophy, muscle weakness, and cardiovascular deconditioning.[15] Among young healthy people, only 1 week of bed rest resulted in important strength losses, with the most profound muscle volume and strength losses occurring in the lower extremities.[16, 17] These effects are more pronounced in older adults.[18] Ten days of bed rest among healthy older volunteers (mean age = 67) documented decreases in overall lean body mass of 3.2%, decreases in lower extremity lean body mass of 6.3%, and declines in knee extensor strength of 1.6% per day.[19] Over a 10 day stay, this is a

cumulative 16% loss of knee extensor strength. Coupled with a higher prevalence of sarcopenia (lower than normal muscularity) in the elderly population (ranging from 7 to over 50%[20-23]), elderly critically ill patients are likely more vulnerable to detrimental effects of bed rest. Given the importance of muscle for functional capacity, loss of muscle has multifactorial effects on critically ill patients. Since patients are most likely receiving bed rest early during their ICU stay, and the 1st week of bed rest is most vulnerable for muscle strength losses,[17] more research is needed to identify rehabilitation interventions to attenuate muscle strength losses during this critical time.

2.2 Rehabilitation in the ICU: improving function, reducing utilization, and saving costs

Early physiotherapy, started while a patient is receiving mechanical ventilation, can improve patient outcomes, reduce ICU and hospital length of stay, and potentially save a hospital millions of dollars.[3-7] To-date, interventions started after an ICU survivor's hospital stay have not improved patient outcomes,[24-26] whereas those started during a patient's ICU stay have, [3, 6] suggesting that initiating rehabilitation early is important. Traditionally, critically ill patients are often perceived as "too sick" to receive rehabilitation.[27] In contrast, a growing body of evidence supports that early ICU rehabilitation is not only safe but effective for both patients and hospitals.[3-6, 28-31] However, as in any emerging field, there are few RCTs of early rehabilitation started in the ICU, and fewer in newly mechanically ventilated patients. A systematic review of early mobilization in the ICU identified 15 reports of 12 unique studies published from 2000-2011.[32] Of the 12 studies, 9 were in the acute ICU setting (vs. a chronic ventilator facility), 4 had a comparison group, and only 2 were RCTs. Since publication of this review, we identified another non-randomized study conducted in the surgical ICU.[33]

Rehabilitation started in the ICU, while a patient receives mechanical ventilation, can improve patient outcomes, reduce ICU and hospital length of stay, and could save hospitals millions of dollars through length of stay (LOS) reductions. Of the available evidence, 5 unique studies (3 non-randomized, 2 randomized) demonstrated promising effects of early rehabilitation[3-7, 29, 33] (**Table 1**). In the 3 non-randomized studies, increased early exposure to rehabilitation improved clinical and utilization outcomes. For example, in a single-center, 57 patient pre-post quality improvement study of PT and OT initiated in patients MV > 4 days in a medical ICU, those in the intervention group received more rehabilitation sessions in ICU (median 7 vs. 1, p <0.001), had higher functional status (defined as sitting or greater mobility, 78% vs. 56%, p=0.03), and had shorter ICU and hospital LOS (ICU=4.9 vs. 7.0 days, p=0.020; Hospital=14.1 vs. 17.2 (p=0.030).[5, 29] **This study projected a conservative annual savings of over $1.2 million dollars for 1 ICU with 900 admissions yearly.**[7]

In a single-center, 193 patient non-randomized trial of augmented PT (4 therapists working in shifts to provide 12 hours weekday coverage plus 8 hours weekend coverage) and protocolized care versus usual PT care (1 therapist providing 8 hours weekday coverage 4.5 days/ week plus 4 ICU patients per weekend) in a surgical ICU, increased PT exposure (1.38 vs. 0.57 sessions per patient per day) reduced extubation failure (6.4% vs. 21%, relative risk reduction for intervention 0.23, 95% confidence interval (CI), 0.05 to 0.98), and trend towards shorter time from ICU to hospital discharge (7.4 vs. 10.5 days, p=0.05).[33] Finally, in the largest ICU rehabilitation study to-date, a single-center, 330 patient non-randomized trial of a 3-person mobility team and 4-phase mobility protocol initiated in a medical ICU within 48 hours of MV admission improved rehabilitation exposure, mobility, and LOS compared to usual care.[4] More patients in the early mobility group received rehabilitation in the ICU (91.4% vs. 12.5%, p≤0.001) and during their hospital stay (80% vs. 45%, p≤0.001). Those receiving early mobility had a shorter time to getting out of bed (8.5 vs. 13.7 days, p<0.001), and shorter adjusted ICU and hospital LOS (ICU=5.5 vs. 6.9 days, p=0.027; hospital=11.2 vs. 14.5 days, p=0.006).

To-date, only 2 RCTs representing 192 patients support the effectiveness of early rehabilitation in the ICU. In a 2-centre, 104 patient RCT of early PT and OT interventions in a medical ICU, 59% of patients receiving early rehabilitation within 1.5 days of MV returned to independent

functional status at hospital discharge, compared to 35% of those receiving therapy within 7.4 days of MV (p=0.02).[6] The main difference between the intervention and control groups was receipt of PT and OT while patients received MV. On average, patients in the intervention group received 20 minutes of rehabilitation daily while mechanically ventilated, whereas those in the control group received no therapy during MV. In a single-centre, 90 patient RCT of in-bed cycle ergometry in a medical-surgical ICU, those randomized to in-bed cycle ergometry and standard PT achieved 196 m in the 6-minute walk test at hospital discharge, compared those receiving standard PT, who achieved 143 m (p<0.05).[3] **Thus, starting rehabilitation while a patient receives MV is feasible, improves patients' function, and may have an important impact on critical care and hospital utilization.**

However, the existing evidence has limitations. Despite emerging evidence supporting early rehabilitation in the ICU, PT and OT is often under-prescribed, occurring on as few as 6% of all ICU days.[34] Of the 3 non-randomized studies, all focused on process measures (e.g., receipt of therapy, utilization), and none were designed or statistically powered to study patient functional outcomes.[4, 5, 29, 33] Methodologically, of the 2 RCTs, the primary outcome measure of 1 RCT was a non-validated composite binary outcome (functional independence = independent ambulation and independence in 6 activities of daily living),[6] and the second may have biased outcome ascertainment because it did not report whether outcomes assessors were blinded to treatment assignment. While the RCTs demonstrated improved patient function, none showed differences in ICU or hospital LOS between the intervention and control groups, whereas the non-randomized studies did improve LOS. To-date, there are no economic evaluations of these RCTs advising whether investments in additional rehabilitation are cost-effective. Results from the 2 RCTs have not been replicated in other ICU settings or in Canada.

Most importantly, of the 2 RCTs, the early rehabilitation occurred in a medical ICU population, and not in a medical-surgical ICU population, which is more common in Canada. In-bed cycling, while studied in a medical-surgical population, did not start until the patients were 14 days into their ICU stay. In our study, we plan to combine the strengths of previous research – offer in-bed cycling early, within the first 4 days of mechanical ventilation.[3] With a projected shortage of PTs in Canada and increased demand for PT services,[35, 36] rigorous evaluation is urgently needed of a structured rehabilitation intervention in the ICU, powered to examine effects on a functional outcome.

2.3 CYCLE: A systematic research program of early rehabilitation in the ICU

While early rehabilitation is gaining clinical popularity, there are important gaps in the field overall: **1) Safety** – while much of the ICU literature reports safety events such as catheter or tube dislodgement,[3, 4, 28, 37] there are few studies documenting critically ill patients' physiologic response to early physical activity during MV; **2) Feasibility** – Since the previous ICU studies occurred in the United States[4-6, 29], Belgium[3], and South Africa[33], little is known about Canadian substitute decision makers' receptiveness to enrolling patients into early rehabilitation clinical trials, which may impact future research feasibility; **3) Replication** – Of the 2 RCTs of early rehabilitation, the frequency, intensity, type, and duration of therapy between the rehabilitation and control groups were incompletely described and controlled.[3, 6] To facilitate future research replication or practice implementation, precise details about the intervention and comparator are needed.[38, 39]

The primary objective of this research program is to design and conduct a structured early rehabilitation intervention in critically ill patients to improve patient-centered outcomes such as physical function and quality of life. The centerpiece of this research is the cycling-themed Critical Care Cycling to Improve Lower Extremity Strength, "**CYCLE**" Program of Research, studying early in-bed cycling initiated in critically ill patients while receiving MV. CYCLE is a 3-phase, 8-project portfolio of studies that will culminate in a phase III, multi-centre RCT and economic evaluation. RCTs are expensive to conduct and need background work to ensure they are optimally designed.[40, 41] This

proposal is part of a comprehensive, rigorous, multidisciplinary research program to inform a future multi-centre RCT (**Figure 1**). Herein we describe the first study in this program: **TryCYCLE**.

2.4 Cycle ergometry: a promising early rehabilitation intervention

In-bed cycle ergometry is an attractive early rehabilitation intervention for MV patients because it can occur in bed, specific details about the intervention can be recorded, and only requires 1 clinician to operate. During in-bed cycling, patients only move their legs, not their head or torso. Thus, we believe there is a low likelihood of unplanned extubation or inadvertent catheter removal. Cycling duration, intensity, and distance are easily recorded to facilitate replication in other settings. Unlike ambulation during MV, which can require up to 4 clinicians,[42] cycling can occur with 1 clinician.[3] A case series published in abstract form of 20 MV patients receiving 20 minutes of passive cycling showed no increase in cardiac output or oxygen consumption, and could occur during vasoactive infusions.[43] However, this study did not appear to report any outcome data beyond 1 treatment session per patient.

In-bed cycling introduced after 2 weeks of ICU stay increased physical function at hospital discharge,[3] and early passive cycling appears safe in MV patients.[43] Both active and passive cycling induce similar cerebral flood flow patterns, thus even passive cycling may be helpful, particularly in sedated patients.[44] In summary, in-bed cycling is a potentially efficacious early rehabilitation intervention for newly MV patients. **To our knowledge, there has been no systematic evaluation of early in-bed cycling in mechanically ventilated patients.** While the use of in-bed cycling initiated 2 weeks into a patient's ICU stay improves patient outcomes at hospital discharge, we do not know if it is safe and feasible to introduce this protocolized intervention earlier in a patient's ICU stay. Before initiating a pilot randomized study of early in-bed cycling in mechanically ventilated patients, a prospective cohort study is needed to determine whether early in-bed cycling can be safely and feasibly delivered. **The purpose of this study is to determine the safety and feasibility of early in-bed cycling in mechanically ventilated patients in ICU.**

3.0 RESEARCH PLAN

3.1 Research Design: We will conduct a single-center, prospective observational cohort pilot study. After informed consent, patients will receive 30 minutes of in-bed cycling *in addition* to routine physiotherapy, 6 days per week, for the duration of their ICU stay. If a patient is re-admitted to ICU during the index hospitalization, we will re-start cycling. During every in-bed cycling session, we will record patients' vital signs, and cycling achievements (described below). At ICU awakening, ICU discharge, and hospital discharge, we will collect physical function measures (described below). **Figure 2** outlines the trial schema.

3.2 Research Ethics Approval, Screening, and Informed Consent: We will seek ethics approval from the Hamilton Integrated Research Ethics board. **Our target population is medical-surgical ICU patients requiring mechanical ventilation, previously independent for mobility before hospital admission. Table 2** outlines the trial inclusion and exclusion criteria. A critical care research coordinator will screen the ICU patient census regularly to identify patients who meet inclusion and exclusion criteria. Because MV is a requirement for study entry, patients will likely be sedated and unable to provide informed consent. In such cases, we will obtain consent from their surrogate decision-maker. Once patients are no longer sedated, they will be evaluated for capacity and consented for continuation in the study.

3.3 Intervention: Patients will receive 30 minutes of in-bed cycling in addition to routine physiotherapy, 6 days per week, for the duration of their ICU stay (to a maximum of 28 days). A

research PT will implement a 2-step process for each research session to ensure it is safe to initiate and continue in-bed cycling.

Step 1: Because of the dynamic nature of critical illness, we will carefully screen patients daily for conditions precluding in-bed cycling. For example, we will not initiate cycling if a patient has cardiac or respiratory instability, active major bleeding, severe agitation, or a new condition interfering with cycling. **Table 3** outlines detailed screening criteria for initiating in-bed cycling. If the patient meets Step 1 criteria, the therapist will coordinate with the patient's bedside nurse for optimal timing of the research session.

Step 2: During every in-bed cycling session, we will carefully monitor patients for safety and indications for termination of cycling, including signs of cardiac or respiratory instability, and catheter or tube dislodgement. **Table 4** outlines detailed safety criteria.

3.3.1 Cycling Details: We will aim for the patient to complete as much active cycling as possible during each session. We will use a specialized cycle ergometer (Restorative Therapies RT300 supine cycle ergometer, **Figure 3**), which attaches to the end of the patient's bed and provides 3 possible cycling modes: passive (i.e., no patient initiation), active-assisted (i.e., partially initiated), or active (i.e., fully initiated), in increasing levels of resistance.[3] Patients will be positioned semi-recumbently[3] per ventilator-associated pneumonia prevention guidelines.[45, 46]

The PT will place the patient's legs in the cycle ergometer, starting with passive cycling at a rate of 20 cycles per minute with no resistance per previous research.[3] If patients initiate active cycling, the PT will increase resistance to promote more active participation and muscle activation. If patients tolerate some resistance, the PT will increase resistance in 3-minute intervals, as tolerated. If the patient stops cycling actively, the ergometer will revert to passive cycling. If this occurs, we will allow the patient to cycle passively for 1 minute at 20 cycles per minute, and we will encourage active cycling through verbal cues, initiation of active-assisted cycling, or reducing the passive cycling rate to 10 cycles per minute. Once patients re-start active cycling, we will titrate resistance as described above. We will record all cycling parameters during every session.

3.4 Data Collection: Potential Confounders, Exposures, and Outcomes

3.4.1 Patient demographics and ICU exposures: We will collect baseline data including patient demographics, reason for ICU admission, medical versus surgical status, severity of illness and pre-hospital physical function. ICU-related variables captured daily during the patient's ICU stay include illness severity, other life supports, drug exposure and nutrition. Further details are in **Tables 5 and 6**.

3.4.2 Cycling Measurements: ICU patients have continuous vital sign monitoring and periodic recording of many other variables as part of routine care. TryCYCLE will capitalize on standardized ICU data collection. For the study, at baseline and post-exercise, the PT will manually record the patient's HR, RR and blood pressure (BP) at 5 minute intervals for 15 minutes. At baseline and at 15 minutes post-cycling, the PT will measure sedation status using the Richmond Agitation and Sedation Scale,[47] presence of ICU delirium using the Confusion and Assessment Method for ICU, [48] and MV settings. During the cycling sessions, the PT will continuously monitor the patient for safety events (**Table 4**), and manually record HR, RR and BP at 5 minute intervals. At the end of the session, the PT will record the total cycling time, duration of active vs. passive cycling, workload, and distance. These TryCYCLE measurements are already routinely collected for physiotherapy documentation in practice.

3.4.3 Physiology Measurements: Variability measures offer an additional evaluation of underlying physiologic state; depressed variability reflects a more stressed system, less ability to compensate, and greater severity of illness in critically ill patients.[49] In addition, altered heart rate variability provides

insight regarding the state of the autonomic nervous system[50] and is commonly used in exercise physiology to assess a person's exercise reponse.[51, 52] In critical care, variability measures of heart rate (HR) and respiratory rate (RR) are emerging as ways of assessing the response to conditions such as sepsis.[49, 53-55] We will explore variability measures before, during, and after physical activity in the ICU.

For the variability measurements, patients will receive additional continuous heart and respiratory monitoring using the Zephyr Bioharness™ (Annapolis, MD), a non-invasive portable data logging system (HR and RR). This is a small device that easily fits around the patient's chest like an exercise monitor. The monitoring will occur for 15 minutes before, during, and 15 minutes after cycling sessions. Data will electronically harvested from the data logger (via USB). The continuously recorded heart rate and respiratory rate waveforms are subsequently able to undergo continuous variability analysis to further characterize the physiologic state of the patient.

3.4.4 Outcomes: Our overall hypothesis is that early in-bed cycling in mechanically ventilated patients is as safe (Objective 1)[3] and feasible (Objective 2) [3, 6]as described in previous research.

Our primary outcome is safety, defined as our ability to deliver 30 minute in-bed cycling sessions without early termination due to changes in a patient's physiologic status (e.g., intolerable dyspnea, desaturation, cardiovascular instability, see **Table 4**). We will determine the feasibility of consent, providing research sessions, and collecting physical outcome measures at ICU awakening, ICU discharge, and hospital discharge. Trained PTs will measure muscle strength (manual muscle strength testing,[56, 57] hand held dynamometry,[58] hand grip dynamometry[57]), function (activities of daily living scale,[59] functional status scale for ICU,[37] and physical function test for ICU[60]). These measures have known reliability in the critical care population and are common outcomes in ongoing ICU rehabilitation studies[61, 62]. We will also explore the feasibility of using the Patient Specific Functional Scale, a patient-rated functional scale with strong psychometric characteristics outside the ICU setting.[63-65] Secondary outcomes also include hospital discharge location (e.g., home vs. rehab), length of MV, ICU and hospital stay, and ICU and in-hospital mortality. **Table 7** outlines our safety, feasibility, and physical outcomes. **Table 8** outlines the measurement characteristics of each of our outcome measures.

3.5 Sample size calculation: **Our sample size calculation is based on the number of a-priori termination events (primary outcome) leading to discontinuation of an in-bed cycling session (see Table 4).** We hypothesize that the observed termination event rate will not be different, or will be better (i.e., lower) than other early rehabilitation studies (0 to 4%).[32] Using a confidence interval (CI) approach for binary proportions, [41] we need to observe 164 in-bed cycling sessions to ensure an observed termination rate is within an upper 95% CI of 3% from a point estimate of 4% (7 events). **Assuming a median ICU length of stay of 7 days given these inclusion and exclusion criteria, and prior studies in this ICU,[66] and considering up to 2 days to enroll patients from ICU admission (total 5 cycling sessions), we will aim to enroll 33 patients.** Based on an estimated 70% consent rate,[6] we will need to identify 43 eligible patients (approximately 5 patients per month for a 9 month study).

3.6 Data management: We will use the iDataFax data management system from the CLARITY methodological support unit at McMaster University. All data will be entered into this secure, web-based platform, which has been used extensively for clinical trials in Canada and internationally (http://www.datafax.com/software/idatafax/).

3.7 Analysis

Objective 1: Safety

a. **Primary Outcome: Termination**: The *a priori* safety event rate is **low** (0 to 4%).
b. **Adverse events**: The line or tube dislodgement rate is **low** (<4%).

Analysis for Objectives 1 a and b: Based on a systematic review of rehabilitation studies conducted in critically ill patients, we expect that safety events are low (e.g., intolerable dyspnea, desaturation, cardiovascular instability, see **Table 4**) according to our pre-specified criteria for terminating research sessions (0 to 4%) [32]. Similarly, based on this systematic review, we expect low event rates for catheter (e.g., central venous catheter, arterial catheter) or tube (e.g., nasogastric tube, see **Table 4**) dislodgement (<4%)[32]. We will calculate the binary proportion and 95% confidence interval for event rates and compare our rates against previous ICU rehabilitation studies. **We hypothesize that our event rates will not be different, or will be better (i.e., lower) than the literature.**

c. **Stability**: Patients **can tolerate in-bed cycling** while critically ill (heart and respiratory rates within 20% of baseline, no change in their variability, and blood pressure within 10% of baseline).

Analysis: We will study stability in 2 ways:

i. **Longitudinal analysis** using vital signs (HR, RR, BP) collected manually at 5-minute intervals by the PTs, we will develop linear regression models where time (categorical) is the primary exposure. From each of the models, we will estimate the difference in the baseline mean value at each of the 5 minute activity time points. **We hypothesize that vital signs will not be different from pre-specified baseline levels (20% for HR, RR, and BP [systolic or diastolic]).**

ii. **Exploratory analysis of the baseline (pre- cycling) and the change in variability (during and post- cycling)** of HR and RR. Variability analysis will be performed by the Continuous Multiorgan Individualized Variability Analysis (CIMVA) software engine (Dynamical Analysis Lab, University of Ottawa). The CIMVA engine will collect and process raw HR and RR data from the patient, eliminate artifact, and create inter-beat and inter-breath interval time series. Using each time series, a comprehensive, repetitive, iterative variability analysis will occur using standard techniques (e.g., Time, Frequency, Time-frequency, Scale invariant (Fractal), and Irregularity domain analyses).[51] Variability analysis reports will be created for all cycling sessions and we will identify the best measures to describe patients' responses. **We hypothesize that there is no change in variability among pre-, during, and post-cycling periods.**

Objective 2: Feasibility

a. **Consent**: Consent rate is **high** (70%).

b. **Intervention delivery**: The daily research session delivery rate is **high** (>80%).

Analysis for objectives 2 a and b: To our knowledge, there are no Canadian studies initiating rehabilitation within 4 days of MV. We will examine the feasibility of enrolling patients into our study by comparing our consent rate with the lowest consent rate in ICU rehabilitation RCTs (70%)[6]. The nature of critical illness means that some patients may not meet our pre-specified requirements to proceed with a research session because they are too unstable (**Table 3**). We will assess our ability to deliver cycling during the ICU stay by comparing our daily delivery rate with that achieved in ICU rehabilitation ICUs (80%).[3, 6] We will calculate the proportion and 95% confidence interval for event rates and compare ours against previous ICU rehabilitation trials. **We hypothesize that our event rates will not be different, or will be better (i.e., higher) than existing literature.**

c. **Outcome measures**: Physical outcome measures can be successfully performed in >80% of assessable patients at ICU awakening, ICU discharge, and hospital discharge.

Analysis: In our future RCT, we intend to measure patients' physical abilities at 3 time points: awakening in the ICU, ICU discharge, and hospital discharge. Because of the complex nature of ICU patients including sedation status and medical stability, we need feasibility data on the ability of trained outcomes assessors to successfully ascertain outcomes throughout the patient's hospital stay. For each of our outcome measures, we will record whether the outcome measurement occurred, the result, the amount of time required to collect the data, and any barriers to data collection. We will

calculate the binary proportion and 95% confidence interval for outcome measure ascertainment event rates for each of the above time points. **We hypothesize that our event rates for outcome measure ascertainment will be 80% or higher for each outcome measure, at each of the 3 time points (ICU awakening, ICU discharge, hospital discharge).**

3.7.1 How data from the Analysis will inform future research: We will use data from our pilot study to inform the design of a future multi-centre pilot RCT, which will ultimately inform a multicentre RCT.[40, 41] Specifically, we will systematically study results from TryCYLE to refine our thresholds for withholding research sessions and termination / safety event criteria. Feasibility data will help us refine our ability to recruit patients, deliver in-bed cycling, and obtain outcome measures.

3.7 Pilot Safety and Feasibility Data:

Implementation of early in-bed cycling is safe and feasible in critically ill, mechanically ventilated patients, in a setting where early rehabilitation is a priority.[5, 29] Two of us (MK, JZ) conducted a retrospective chart review of 185 patients who received in-bed cycling over 1.5 years of routine clinical use in the Johns Hopkins Medical ICU. Of 504 patients who received PT, 185(37%) received a total of 561 PT sessions with in-bed cycling (median [IQR] cycling sessions per patient 2[1,4]). The median [IQR] time from MICU admission to first PT session was 5[2,10] days, and from MICU admission to first cycling session was 5[2,10] days. On the days in which patients received in-bed cycling, 447(80%) received mechanical ventilation (280(63%) via oral endotracheal tube, 167(37%) via tracheostomy), 47(8%) received a continuous vasopressor infusion, and 36(6%) received continuous renal replacement therapy. One pre-defined safety event occurred during cycling sessions (0.1% event rate, with 95% upper confidence limit =1.0%): dislodgement of a radial arterial catheter. **Table 9** summarizes patient characteristics and in-bed cycling details.[67]

These preliminary data from an ICU with an in-bed cycle ergometer demonstrates several important aspects of early in-bed cycling: it can occur within the first few days of ICU admission, while patients are receiving MV and other life support measures, and safety events occurred infrequently. The sample size is more than twice that of the published in-bed cycling RCT, which enrolled 90 patients[3]. However, these data have weaknesses consistent with retrospective data collection.[68, 69] Of 561 sessions, 2% did not document cycling duration, of the remaining sessions, the duration of in-bed cycling varied from 1 to 50 minutes, and patient workload was not documented in 20% of all sessions. Moreover, while these data demonstrate safety and feasibility, there was no standardized cycling protocol, no control group, and no reported patient-important outcome measures.

Outcome measure ascertainment is feasible in assessable patients at ICU awakening, ICU discharge, and hospital discharge in a RCT of early neuromuscular electrical stimulation (NMES) in mechanically ventilated patients. The PI of this study (MK) led the NMES study at Johns Hopkins, where PTs conducted outcome measures at the same time points of TryCYCLE.[61] To-date, there are no missing data for serial measures of muscle strength at ICU awakening, ICU discharge and hospital discharge in assessable patients (n=27, unpublished data).

3.8 Rationale for single center study:

A single-center observational study is the best design for this protocol because fundamental data are first needed about the safety and feasibility of in-bed cycling among newly mechanically ventilated patients; a randomized trial is premature at this time. Despite RCT evidence that early rehabilitation within 1.5 days of MV and that in-bed cycling started 14 days after ICU admission improved patients' outcomes at hospital discharge, to-date, no studies initiated in-bed cycling during mechanical ventilation early in the patient's ICU stay. **Moreover, we are introducing new technology for rigorous evaluation into a Canadian ICU.** The main challenges to providing early ICU rehabilitation in Canada are limited human resources and equipment, lack of protocols to guide therapy,

and significant gaps in clinician knowledge and training to safely provide rehabilitation.[70] Unlike many prevalent ICU interventions (e.g., mechanical ventilation, thromboprophylaxis), the use of cycle ergometry in Canadian ICUs is uncommon. In a recent national survey led by one of us (KK) of Canadian critical care PTs (n=117) and physicians (n=194), only 3% stated that cycle ergometers were frequently or routinely used.[70] The PI of TryCYCLE (MK) is a PT with 14 years of physiotherapy experience and 2.5 years of experience using the cycle ergometer with MV patients in the Johns Hopkins Medical ICU. She will mentor the research PTs in use of the cycle ergometer and conduct of outcome measures with critically ill patients.

3.9 Strengths and Limitations:

TryCYCLE has limitations, including a small sample size. Data from these patients may not be representative of the larger mechanically ventilated population, and the small sample size without a control group precludes any adjusted analysis. However randomization and recruitment of many patients is premature at this early stage of technology evaluation – hence our focus is on safety and feasibility first. Second, in-bed cycle ergometry is not a functional activity and only targets the lower extremities, whereas the upper extremities and torso also weaken with bed rest.[17] Third, given the predicted shortage of PTs in Canada,[73] therapists' time to facilitate this cycling protocol will have an opportunity cost versus other functionally-oriented activities they engage in; nevertheless, PT leadership delivering these cycling sessions is central to their professional development, widens the scope of their rehabilitation tool kit, and secures PT leadership in the future CYCLE research program.

Numerous strengths of this proposed research include the innovative, portable, publically familiar intervention, the unique pilot project to inform future work, the prospective design, multidisciplinary team, and collection of necessary safety and feasibility data to inform a future larger pilot RCT. In both active and passive cycle ergometry, despite differences in muscle activation, there are similar brain activation patterns in the primary somatosentory and pre-motor cortex measured by cerebral blood flow.[44] Thus, in addition to physical benefits, cycle ergometry may impact cognitive function, which is often impaired in ICU survivors.[71] Indeed, passive cycle ergometry in patients with Parkinson's disease improved executive functioning measured by the Trail-making test.[72] This project will allow us to develop specific training materials and standard operating procedures for use of the ergometer. Research PTs will develop expertise to train others for the future multicentre trial.

4.0 RESEARCH TEAM

Our team is uniquely qualified to design, implement, and analyze this project. We are a multidisciplinary team of physiotherapists (MK, JZ), intensivists (KK, JR, AS, MH, DC), respiratory therapists (TP, FC), an exercise physiologist (MM) and a surgeon (AS), from 6 institutions (McMaster University (MK, JR, TP, FC, DC), University of Waterloo (MM), University of Western Ontario (KK), University of Ottawa (AS), Johns Hopkins University (JZ), and University of Toronto (MH)), across academic ranks (Lecturer (JZ, TP), Assistant Professor (MK, KK, MM, JR), Associate Professor (MH, AS), and Professor (DC)). We care for critically ill patients (MK, KK, JR, AS, MH, DC, TP, JZ) and have expertise in rehabilitation of critically ill patients (MK, JZ, KK, MH), advanced exercise physiology (MM, AS), and clinical epidemiology or public health (MK, KK, DC, MH). Below, we outline our anticipated contributions:

4.1 FEASIBILITY: RESEARCH TEAM MEMBERS

1. **Michelle Kho, PT, PhD** (Principal Applicant) has the most clinical experience in Canada with in-bed cycle ergometry with critically ill, mechanically ventilated patients. She held a CIHR Fellowship and Bisby Prize (2010-2012) to lead an RCT of early muscle stimulation (NCT100709124) in the Johns Hopkins University ICU.[61] She is ideally suited to lead this project

from her clinical ICU background, clinical trial leadership experience, and 2 first-author ICU rehabilitation articles.[61, 74] **She will lead the project, has primary responsibility for the intellectual direction of the research, and will be accountable for the reporting and achievement of deliverables. Dr. Kho will recruit and train research physiotherapists and outcome assessors. Commitment**: 10 h/wk.

2. **Karen Koo, MD, MSc** leads a clinical research program, *Acute Rehabilitation in Canadian ICUs*, and recently led a national survey of physicians and physiotherapists on the knowledge, perspectives, and stated practice of acute rehabilitation in Canadian ICUs[70, 75]. **She will contribute to the research from a design and implementation perspective based on results from her survey work.** Together, she and Dr. Kho have collaborated on 1 peer-review publication, and 1 funded project on early mobilization in the ICU. **Commitment**: 4 h/week
3. **Deborah Cook, MD, MSc** is an international leader in the design, implementation, analysis, and dissemination of rigorous clinical trials in the ICU. **She brings expertise in critical care and research methodology and will help address interdisciplinary facilitators and barriers to implementation of the intervention.** Together, she and Dr. Kho have co-authored 12 peer-review publications since 2005, primarily in critical care medicine methodology and psychometrics. **Commitment**: 2 h/week.
4. **Margaret Herridge, MD, MPH** brings world-renowned expertise in the area of ICU-acquired weakness, functional outcomes and quality of life in her landmark work on survivors of ARDS (*NEJM*)[11, 12] and SARS (*Arch Int Med*). As the first investigator in the world to methodically and comprehensively investigate survivors of critical illness, she is now pioneering Canada's CIHR-funded multi-center Towards Recover research program, dedicated to understanding the needs of survivors of prolonged mechanical ventilation and their family caregivers, while constructing a formal rehabilitation program for them. **She will provide expertise in protocol development and outcome measures. Commitment:** 2 h/wk.
5. **Marina Mourtzakis, PhD** studies the interactions of body composition, nutrition, exercise, and metabolism in health and disease with a specific focus on cancer and critical care. Her current research examines the clinical outcomes of muscle atrophy in cancer and critical care and investigates the effects on protein and glucose metabolism in these populations. **She will contribute to the research from an exercise physiology perspective. Commitment**: 2 h/wk.
6. **Andrew Seely, MD, PhD** is the Director of the Dynamical Analysis Lab at the University of Ottawa. Dr. Seely has expertise applying complex systems science, particularly use of variability measurements, to the care and understanding of critically ill patients.[49, 51, 76-79] **He will contribute substantively from critical care medicine and surgical perspectives, and use and implementation of variability measures during exercise in the ICU.** He will mentor 2 graduate students for the analysis of variability data (see budget justification). **Commitment**: 3 h/wk.
7. **Jill Rudkowski, MD** is the ICU Clinician Educator at St. Joseph's Healthcare with an interest in research improving patient outcomes. She is the Site Investigator for the Towards Recover prospective longitudinal cohort study (see Margaret Herridge). **She will provide input from an end-user perspective regarding the design and implementation of the protocol, helping to oversee issues of safety and feasibility. Commitment**: 1.5 h/wk.

COLLABORATORS

1. **Jennifer Zanni, PT, DSc(PT)** has over 14 years of experience in the acute care setting and is a Clinical Specialist in the Medical ICU at JHH. Dr. Zanni has over 4 years of clinical experience with in-bed cycle ergometry and is a clinical leader for physical therapists implementing early rehabilitation in the ICU setting. **She will provide input from an end-user perspective regarding the design and implementation of the protocol and clinical decision making for the**

intervention. Together, she and Dr. Kho have co-authored 2 peer-review publications in early rehabilitation in ICU patients. **Commitment**: 1.0 h/wk.

2. **Thomas Piriano, RRT** is the Best Practices Clinical Educator for Respiratory Therapy at St. Joseph's Healthcare and Lecturer at McMaster University. He has over 10 years of experience in respiratory therapy, all in critical care. **He will provide input regarding mechanical ventilator management of patients while they are receiving cycling, and provide input from an end-user perspective regarding the implementation of the protocol from a respiratory therapist perspective. Commitment**: 1.0 h/wk.
3. **France Clarke, RRT** is a full time Research Coordinator with 11 years of experience screening, consenting, enrolling and coordinating international critical care clinical trials with Dr. Deborah Cook at St. Joseph's Healthcare. Clinically, she is trained as a Registered Respiratory Therapist. **She will screen, enroll, perform data collection, implement the protocol, and coordinate the project overall**. **Commitment**: 16 h/wk.

4.2 ENVIRONMENT AND RESEARCH SUPPORT

4.2.1 Clinical research setting - This project will be conducted in the **ICU at St. Joseph's Healthcare**, Charlton Site, Hamilton, ON (see support letter). It has a 20-bed medical-surgical ICU, receives 1,000 ICU admissions annually, and is recognized as a world leader in critical care clinical research. It has an established research program under the leadership of Dr. Deborah Cook and is the clinical home for Drs. Kho, Cook, Rudkowski, Mr. Piriano, and Ms. Clarke. Unlike other Canadian ICUs who report limited access to cycle ergometry[70], this ICU has a dedicated bedside cycle ergometer and dedicated outcome measure equipment for this study (e.g., hand grip dynamometer, hand-held dynamometer).

4.2.2 We will use the CLARITY methodological support unit from McMaster University. Dr. Cook (co-investigator, please see above) is a core faculty member in CLARITY, having established relationships with database developers (i.e., iDataFax) and biostatisticians. She successfully coordinated an international, 67 center, 3,764 patient critical care venous thromboembolism prophylaxis RCT through CLARITY.[66] CLARITY has extensive experience developing research databases, conducting critical care clinical trials, and analyzing complex databases..

4.2.3 Canadian Critical Care Trials Group (CCCTG) – Five team members (Kho, Koo, Seely, Herridge, Cook) are members of the CCCTG, a multidisciplinary group of Canadian investigators committed to conducting high-quality studies in Canada and globally. Landmark research led by one of our team members (Herridge)[11, 12] conducted by CCCTG investigators led to heightened awareness of physical impairments and poor quality of life of ICU survivors. The CYCLE research program is enthusiastically endorsed by the CCCTG, which will facilitate protocol development, problem-solve as necessary and aid in the future expansion of this research program across Canada and internationally.

4.2.4 Training Highly Qualified Personnel - This pilot project is ideal for training highly qualified personnel. The enabling, coordination, monitoring, and analysis of the data collection for variability analysis (Objective 1c(i)), Safety), will require a **Master's level biomedical engineer** and a **PhD level biomedical engineer** under the supervision of Dr. Andrew Seely. Trainees will attend the 1-day, in-person start-up meeting in Hamilton, and have the opportunity to observe the research protocol operationalized in the clinical ICU setting (see Budget Justification).

4.3 KNOWLEDGE TRANSLATION PLAN AND RESEARCH DELIVERABLES

Knowledge from our single centre pilot study is crucial to the design and conduct of our future full-scale trial. We will use materials generated from this study for future data training, recruitment, and promotion of our future full-scale trial. Following completion of our study, we will identify future participating sites for the next phase of our research program. **Table 10** details specific research receptors and the types of knowledge we will share with these groups.

We will communicate our safety results locally, nationally, and internationally. Locally, we will present results at Critical Care and Rehabilitation multidisciplinary grand rounds in our respective institutions. Nationally, we will share safety results at clinical conferences, such as the Critical Care Canada Forum, an annual multidisciplinary meeting for ICU clinicians. Internationally, we will communicate safety results through presentations at rehabilitation (e.g., World Congress of Physiotherapy), critical care (e.g., American Thoracic Society, Society for Critical Care Medicine), and complex systems (e.g., International Conference on Complexity in Acute Illness) meetings. We will publish results in open access peer-review journals (or purchase open access rights), facilitating international dissemination of this emerging research program of high interest to the global community.

4.4 RESEARCH TIMELINE

	2013	2014					
**Item**	**October - December**	**January – February**	**March - April**	**May - June**	**July - August**	**September - October**	**November - December**
**Preparation**							
Funding Start Date October 2013							
REB application and approval							
Develop case report forms							
Develop research database							
Train research physiotherapists							
In-person start-up meeting							
**Execution**							
Enrolment							
Data collection and data entry							
Data cleaning							
Student training/ mentorship							
**Analysis**							
Data analysis							
Manuscript preparation							
Abstract submission to international conferences							
Manuscript submission							
Presentation at national & international scientific meetings							2015

5.0 SIGNIFICANCE

By 2026, the number of patients in Canada aged >60 years requiring MV is expected to increase by 105%,[13] presenting an urgent need to proactively address their rehabilitation needs. Adults > 65 years of age are less likely enrolled in critical care trials.[80] In-bed cycling is an attractive intervention because it can occur with 1 clinician, can be protocolized, has proven effectiveness in long-stay ICU patients[3] and improved cognition in patients with neurological disease.[72] Our modest request to collect pilot data will accelerate key safety and feasibility information for the cycling research program, particularly the phase 3 RCT we plan. If effective, early in-bed cycling could save in-patient hospitalization costs and improve quality of life for ICU survivors.

REFERENCES

1. Misak CJ. ICU-acquired weakness: obstacles and interventions for rehabilitation. Am J Respir Crit Care Med 2011;183:845-6.
2. Iwashyna TJ, Ely EW, Smith DM, Langa KM. Long-term cognitive impairment and functional disability among survivors of severe sepsis. JAMA 2011;304:1787-94.
3. Burtin C, Clerckx B, Robbeets C, et al. Early exercise in critically ill patients enhances short-term functional recovery. Crit Care Med 2009;37:2499-505.
4. Morris PE, Goad A, Thompson C, et al. Early intensive care unit mobility therapy in the treatment of acute respiratory failure. Crit Care Med 2008;36:2238-43.
5. Needham DM, Korupolu R, Zanni JM, et al. Early physical medicine and rehabilitation for patients with acute respiratory failure: a quality improvement project. Arch Phys Med Rehabil 2010;91:536-42.
6. Schweickert WD, Pohlman MC, Pohlman AS, et al. Early physical and occupational therapy in mechanically ventilated, critically ill patients: a randomised controlled trial. Lancet 2009;373:1874-82.
7. Lord RK, Mayhew CR, Korupolu R, et al. ICU Early Physical Rehabilitation Programs: Financial Modeling of Cost Savings. Crit Care Med 2013.
8. Leeb K, Jokovic A, Sandhu M, Zinck G. Intensive care in Canada. Healthc Q 2006;9:32-3.
9. Erickson SE, Martin GS, Davis JL, Matthay MA, Eisner MD. Recent trends in acute lung injury mortality: 1996-2005. Crit Care Med 2009;37:1574-9.
10. Spragg RG, Bernard GR, Checkley W, et al. Beyond mortality: future clinical research in acute lung injury. Am J Respir Crit Care Med 2010;181:1121-7.
11. Herridge MS, Cheung AM, Tansey CM, et al. One-year outcomes in survivors of the acute respiratory distress syndrome. N Engl J Med 2003 348:683-93.
12. Herridge MS, Tansey CM, Matte A, et al. Functional disability 5 years after acute respiratory distress syndrome. N Engl J Med 2011;364:1293-304.
13. Needham DM, Bronskill SE, Calinawan JR, Sibbald WJ, Pronovost PJ, Laupacis A. Projected incidence of mechanical ventilation in Ontario to 2026: Preparing for the aging baby boomers. Crit Care Med 2005;33:574-9.
14. Barnato AE, Albert SM, Angus DC, Lave JR, Degenholtz HB. Disability among elderly survivors of mechanical ventilation. Am J Respir Crit Care Med 2011;183:1037-42.
15. Brower RG. Consequences of bed rest. Crit Care Med 2009;37:S422-8.
16. Ferrando AA, Stuart CA, Brunder DG, Hillman GR. Magnetic resonance imaging quantitation of changes in muscle volume during 7 days of strict bed rest. Aviat Space Environ Med 1995;66:976-81.
17. LeBlanc AD, Schneider VS, Evans HJ, Pientok C, Rowe R, Spector E. Regional changes in muscle mass following 17 weeks of bed rest. J Appl Physiol 1992;73:2172-8.
18. Kortebein P, Symons TB, Ferrando A, et al. Functional impact of 10 days of bed rest in healthy older adults. J Gerontol A Biol Sci Med Sci 2008;63:1076-81.
19. Kortebein P, Ferrando A, Lombeida J, Wolfe R, Evans WJ. Effect of 10 days of bed rest on skeletal muscle in healthy older adults. JAMA 2007;297:1772-4.
20. Baumgartner RN, Koehler KM, Gallagher D, et al. Epidemiology of sarcopenia among the elderly in New Mexico. Am J Epidemiol 1998;147:755-63.
21. Janssen I, Heymsfield SB, Ross R. Low relative skeletal muscle mass (sarcopenia) in older persons is associated with functional impairment and physical disability. J Am Geriatr Soc 2002;50:889-96.

22. Melton LJ, 3rd, Khosla S, Crowson CS, O'Connor MK, O'Fallon WM, Riggs BL. Epidemiology of sarcopenia. J Am Geriatr Soc 2000;48:625-30.
23. Newman AB, Kupelian V, Visser M, et al. Sarcopenia: alternative definitions and associations with lower extremity function. J Am Geriatr Soc 2003;51:1602-9.
24. Cuthbertson BH, Rattray J, Campbell MK, et al. The PRaCTICaL study of nurse led, intensive care follow-up programmes for improving long term outcomes from critical illness: a pragmatic randomised controlled trial. BMJ 2009;339:b3723.
25. Elliott D, McKinley S, Alison J, et al. Health-related quality of life and physical recovery after a critical illness: a multi-centre randomised controlled trial of a home-based physical rehabilitation program. Crit Care 2011;15:R142.
26. Jones C, Skirrow P, Griffiths RD, et al. Rehabilitation after critical illness: a randomized, controlled trial. Crit Care Med 2003;31:2456-61.
27. Gosselink R, Bott J, Johnson M, et al. Physiotherapy for adult patients with critical illness: recommendations of the European Respiratory Society and European Society of Intensive Care Medicine Task Force on Physiotherapy for Critically Ill Patients. Intensive Care Med 2008;34:1188-99.
28. Bailey P, Thomsen GE, Spuhler VJ, et al. Early activity is feasible and safe in respiratory failure patients. Crit Care Med 2007;35:139-45.
29. Needham DM, Korupolu R. Rehabilitation quality improvement in an intensive care unit setting: implementation of a quality improvement model. Top Stroke Rehabil 2010;17:271-81.
30. Perme C, Chandrashekar R. Early mobility and walking program for patients in intensive care units: creating a standard of care. Am J Crit Care 2009;18:212-21.
31. Thomsen GE, Snow GL, Rodriguez L, Hopkins RO. Patients with respiratory failure increase ambulation after transfer to an intensive care unit where early activity is a priority. Crit Care Med 2008;36:1119-24.
32. Adler J, Malone D. Early mobilization in the intensive care unit: a systematic review. Cardiopulm Phys Ther J 2012;23:5-13.
33. Hanekom SD, Louw Q, Coetzee A. The way in which a physiotherapy service is structured can improve patient outcome from a surgical intensive care: A controlled clinical trial. Crit Care 2013;16:R230.
34. Needham DM, Dowdy DW, Mendez-Tellez PA, Herridge MS, Pronovost PJ. Studying outcomes of intensive care unit survivors: measuring exposures and outcomes. Intensive Care Med 2005;31:1153-60.
35. Landry MD, Hamdan E, Al Mazeedi S, Brooks D. The precarious balance between 'supply' and 'demand' for health care: the increasing global demand for rehabilitation service for individuals living with chronic obstructive pulmonary disease. Int J Chron Obstruct Pulmon Dis 2008;3:393-6.
36. Landry MD, Jaglal S, Wodchis WP, Raman J, Cott CA. Analysis of factors affecting demand for rehabilitation services in Ontario, Canada: a health-policy perspective. Disabil Rehabil 2008;30:1837-47.
37. Zanni JM, Korupolu R, Fan E, et al. Rehabilitation therapy and outcomes in acute respiratory failure: an observational pilot project. J Crit Care 2010;25:254-62.
38. Boutron I, Moher D, Altman DG, Schulz KF, Ravaud P. Extending the CONSORT statement to randomized trials of nonpharmacologic treatment: explanation and elaboration. Ann Intern Med 2008;148:295-309.
39. Hildebrand MW, Host HH, Binder EF, et al. Measuring treatment fidelity in a rehabilitation intervention study. Am J Phys Med Rehabil 2012;91:715-24.
40. Arnold DM, Burns KE, Adhikari NK, Kho ME, Meade MO, Cook DJ. The design and interpretation of pilot trials in clinical research in critical care. Crit Care Med 2009;37:S69-74.

41. Thabane L, Ma J, Chu R, et al. A tutorial on pilot studies: the what, why and how. BMC Med Res Methodol 2010;10:1.
42. Needham DM, Truong AD, Fan E. Technology to enhance physical rehabilitation of critically ill patients. Crit Care Med 2009;37:S436-41.
43. Pires-Neto RC. An Early Trial Of Passive Cycling Exercise In Intensive Care Unit (ICU) Patients. Safety, Physiological and Metabolic Aspects. Am J Respir Crit Care Med 2012;185:A3070.
44. Christensen LO, Johannsen P, Sinkjaer T, Petersen N, Pyndt HS, Nielsen JB. Cerebral activation during bicycle movements in man. Exp Brain Res 2000;135:66-72.
45. Muscedere J, Dodek P, Keenan S, Fowler R, Cook D, Heyland D. Comprehensive evidence-based clinical practice guidelines for ventilator-associated pneumonia: prevention. J Crit Care 2008;23:126-37.
46. Sinuff T, Muscedere J, Cook D, Dodek P, Heyland D. Ventilator-associated pneumonia: Improving outcomes through guideline implementation. J Crit Care 2008;23:118-25.
47. Ely EW, Truman B, Shintani A, et al. Monitoring sedation status over time in ICU patients: reliability and validity of the Richmond Agitation-Sedation Scale (RASS). JAMA 2003;289:2983-91.
48. Ely EW, Inouye SK, Bernard GR, et al. Delirium in mechanically ventilated patients: validity and reliability of the confusion assessment method for the intensive care unit (CAM-ICU). JAMA 2001;286:2703-10.
49. Seely AJ, Christou NV. Multiple organ dysfunction syndrome: exploring the paradigm of complex nonlinear systems. Crit Care Med 2000;28:2193-200.
50. Akselrod S, Gordon D, Ubel FA, Shannon DC, Berger AC, Cohen RJ. Power spectrum analysis of heart rate fluctuation: a quantitative probe of beat-to-beat cardiovascular control. Science 1981;213:220-2.
51. Bravi A, Longtin A, Seely AJ. Review and classification of variability analysis techniques with clinical applications. Biomed Eng Online 2011;10:90.
52. Armstrong RG, Kenny GP, Green G, Seely AJ. Diurnal variation in heart rate variability before and after maximal exercise testing. Chronobiol Int 2011;28:344-51.
53. Ahmad S, Ramsay T, Huebsch L, et al. Continuous multi-parameter heart rate variability analysis heralds onset of sepsis in adults. PLoS One 2009;4:e6642.
54. Bravi A, Green G, Longtin A, Seely AJ. Monitoring and Identification of Sepsis Development through a Composite Measure of Heart Rate Variability. PLoS One 2012;7:e45666.
55. Buchan CA, Bravi A, Seely AJ. Variability analysis and the diagnosis, management, and treatment of sepsis. Curr Infect Dis Rep 2012;14:512-21.
56. Fan E, Ciesla ND, Truong AD, Bhoopathi V, Zeger SL, Needham DM. Inter-rater reliability of manual muscle strength testing in ICU survivors and simulated patients. Intensive Care Med 2010;36:1038-43.
57. Hermans G, Clerckx B, Vanhullebusch T, et al. Interobserver agreement of Medical Research Council sum-score and handgrip strength in the intensive care unit. Muscle Nerve 2012;45:18-25.
58. Vanpee G, Segers J, Van Mechelen H, et al. The interobserver agreement of handheld dynamometry for muscle strength assessment in critically ill patients. Crit Care Med 2011;39:1929-34.
59. Katz S, Ford AB, Moskowitz RW, Jackson BA, Jaffe MW. Studies of Illness in the Aged. The Index of Adl: A Standardized Measure of Biological and Psychosocial Function. JAMA 1963;185:914-9.
60. Skinner EH, Berney S, Warrillow S, Denehy L. Development of a physical function outcome measure (PFIT) and a pilot exercise training protocol for use in intensive care. Crit Care Resusc 2009;11:110-5.

61. Kho ME, Truong AD, Brower RG, et al. Neuromuscular Electrical Stimulation for Intensive Care Unit-Acquired Weakness: Protocol and Methodological Implications for a Randomized, Sham-Controlled, Phase II Trial. Phys Ther 2012;92:1564-79.
62. Parry SM, Berney S, Koopman R, et al. Early rehabilitation in critical care (eRiCC): functional electrical stimulation with cycling protocol for a randomised controlled trial. BMJ Open 2012;2.
63. Chatman AB, Hyams SP, Neel JM, et al. The Patient-Specific Functional Scale: measurement properties in patients with knee dysfunction. Phys Ther 1997;77:820-9.
64. Fairbairn K, May K, Yang Y, Balasundar S, Hefford C, Abbott JH. Mapping Patient-Specific Functional Scale (PSFS) items to the International Classification of Functioning, Disability and Health (ICF). Phys Ther 2012;92:310-7.
65. Horn KK, Jennings S, Richardson G, Vliet DV, Hefford C, Abbott JH. The patient-specific functional scale: psychometrics, clinimetrics, and application as a clinical outcome measure. J Orthop Sports Phys Ther 2012;42:30-42.
66. Cook D, Meade M, Guyatt G, et al. Dalteparin versus unfractionated heparin in critically ill patients. N Engl J Med 2011;364:1305-14.
67. Kho ME, Martin RA, Toonstra AL, Zanni JM, Needham DM. Le Tour De ICU: Feasibility And Safety Of Routine Use Of In-Bed Cycling For Physical Rehabilitation In The Intensive Care Unit. Am J Respir Crit Care Med 2013;proceedings in press.
68. Allison JJ, Wall TC, Spettell CM, et al. The art and science of chart review. Jt Comm J Qual Improv 2000;26:115-36.
69. Worster A, Haines T. Advanced statistics: understanding medical record review (MRR) studies. Acad Emerg Med 2004;11:187-92.
70. Koo K. Survey of Acute Rehabilitation in Canadian Intensive Care Units. Hamilton, ON: McMaster University; 2012.
71. Jackson JC, Mitchell N, Hopkins RO. Cognitive functioning, mental health, and quality of life in ICU survivors: an overview. Crit Care Clin 2009;25:615-28, x.
72. Ridgel AL, Kim CH, Fickes EJ, Muller MD, Alberts JL. Changes in executive function after acute bouts of passive cycling in Parkinson's disease. J Aging Phys Act 2011;19:87-98.
73. Landry MD, Raman S, Al-Hamdan E. Accessing timely rehabilitation services for a global aging society? Exploring the realities within Canada's universal health care system. Curr Aging Sci 2010;3:143-50.
74. Kho ME, Damluji A, Zanni JM, Needham DM. Feasibility and observed safety of interactive video games for physical rehabilitation in the intensive care unit: a case series. J Crit Care 2012;27:219 e1-6.
75. Koo KKY, Choong K, Cook DJ, et al. Development of a Canadian survey of mobilization of critically ill patients in intensive care units: Current knowledge, perspectives and practices. Am J Respir Crit Care Med 2011;183:A3145.
76. Armstrong RG, Ahmad S, Seely AJ, Kenny GP. Heart rate variability and baroreceptor sensitivity following exercise-induced hyperthermia in endurance trained men. Eur J Appl Physiol 2011;112:501-11.
77. Bradley B, Green GC, Batkin I, Seely AJ. Feasibility of continuous multiorgan variability analysis in the intensive care unit. J Crit Care 2012;27:218 e9-20.
78. Seely AJ, Green GC, Bravi A. Continuous Multiorgan Variability monitoring in critically ill patients--complexity science at the bedside. Conf Proc IEEE Eng Med Biol Soc 2012;2011:5503-6.
79. Seely AJ, Macklem PT. Complex systems and the technology of variability analysis. Crit Care 2004;8:R367-84.

80. Cooke CR, Erickson SE, Watkins TR, Matthay MA, Hudson LD, Rubenfeld GD. Age-, sex-, and race-based differences among patients enrolled versus not enrolled in acute lung injury clinical trials. Crit Care Med 2010;38:1450-7.
81. Pohlman MC, Schweickert WD, Pohlman AS, et al. Feasibility of physical and occupational therapy beginning from initiation of mechanical ventilation. Crit Care Med 2010;38:2089-94.
82. Charlson ME, Pompei P, Ales KL, MacKenzie CR. A new method of classifying prognostic comorbidity in longitudinal studies: development and validation. J Chronic Dis 1987;40:373-83.
83. Groll DL, To T, Bombardier C, Wright JG. The development of a comorbidity index with physical function as the outcome. J Clin Epidemiol 2005;58:595-602.
84. Heyland DK, Groll D, Caeser M. Survivors of acute respiratory distress syndrome: relationship between pulmonary dysfunction and long-term health-related quality of life. Crit Care Med 2005;33:1549-56.
85. Knaus WA, Draper EA, Wagner DP, Zimmerman JE. APACHE II: a severity of disease classification system. Crit Care Med 1985;13:818-29.
86. Vincent JL, Moreno R, Takala J, et al. The SOFA (Sepsis-related Organ Failure Assessment) score to describe organ dysfunction/failure. On behalf of the Working Group on Sepsis-Related Problems of the European Society of Intensive Care Medicine. Intensive Care Med 1996;22:707-10.
87. Kleyweg RP, van der Meche FG, Schmitz PI. Interobserver agreement in the assessment of muscle strength and functional abilities in Guillain-Barre syndrome. Muscle Nerve 1991;14:1103-9.

Appendix: TABLES, FIGURES

Table 1: Summary of Selected ICU Rehabilitation Studies (adapted from Kho et al.[61])

Author/Year/Design/ N (analyzed/enrolled)	Population	Intervention (N analyzed/ enrolled)	Comparison (N analyzed/ enrolled)	Timing & Results of Primary Outcome (Intervention vs. Comparison)
Needham 2010[5, 7, 29] Before-after (57/57)	Mechanically ventilated >4 days, cognitively intact without neuromuscular disease	Culture change (4 months): 7 items including (1) default activity orders; (2) change in sedation practice; (3) guidelines for PT and OT consultation; (4) safety events; (5) PT and OT staffing changes (30/30)	Before: 3 months, standard care (27/27)	In ICU (no primary outcome specified) (i) Decrease in benzodiazepine (p=0.03) and narcotics (p=0.05) administration with no change in pain (p=0.79); (ii) Increased PT and OT consults from 70% to 93% (p=0.04)
Hanekom 2013[33] Non-Randomized convenience sample (193/193)	Consecutive patients admitted to a 10-bed surgical ICU	4 research therapists; 5 days coverage 12 hours/ weekday; 8 hours over weekends; management per evidence-based physiotherapy protocol (96/96);	Usual care: 1 therapist, 4.5 days ICU coverage 8 hours/ week day; 4 ICU patients over weekends; management per therapist's clinical decision (97/97)	In ICU / hospital discharge (no primary outcome specified) (i) Increased physiotherapy exposure (1.38 vs. 0.57 sessions per patient per day); (ii) Reduced extubation failure (6.4% vs. 21%, RRR for intervention 0.23, 95%CI, 0.05 to 0.98); (iii) Trend towards shorter time from ICU to hospital discharge (7.4 vs. 10.5 days, p=0.05)
Morris 2008 [4] Non-Randomized (280/330)	Acute respiratory failure	Physician order for early mobility team (145/165)	Usual care (135/165)	In ICU % pts receiving PT: 80% vs. 47% (p<0.001)
Schweickert 2009 [6, 81] RCT (104/104)	Mechanically ventilated <72 h with baseline functional independence	Early PT and OT (49/49)	Usual care (55/55)	Hospital discharge Functional independence: 59% vs. 35% (p=0.02)
Burtin 2009 [3] RCT (52/90)	Critically ill patients with expected prolonged ICU stay (84% mechanically ventilated at enrollment)	Bedside cycle ergometer + customized respiratory PT and standardized PT (26/45)	Customized respiratory PT and standardized PT (26/45)	Hospital discharge 6 minute walk (median [IQR]): 196 m [126-329 m] vs. 143 m [37-226] (p<0.05)

Legend: *PT=physiotherapy; OT=occupational therapy; ICU= intensive care unit; RRR=relative risk reduction; CI=confidence interval; RCT= randomized clinical trial; IQR= interquartile range.*

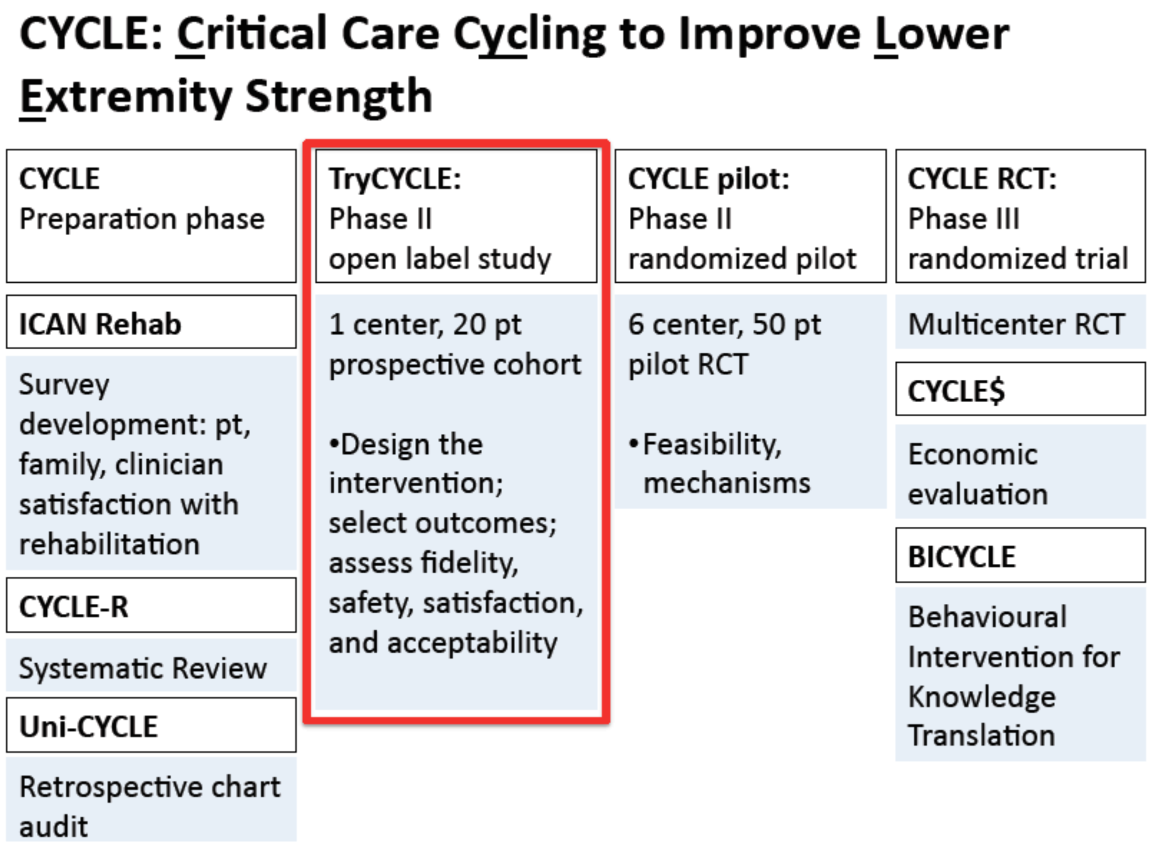

Figure 1: Overview of the CYCLE research program and the role of TryCYCLE in the overall research program.

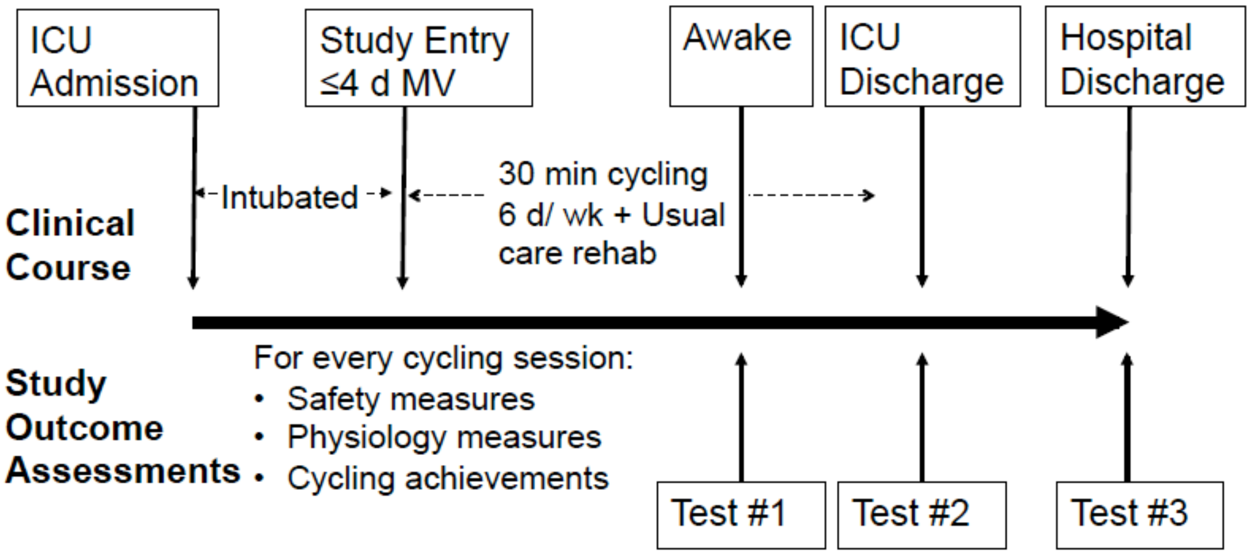

Figure 2: **Study schema**. *Legend*: ICU=intensive care unit; MV=Mechanical ventilation; d=days; wk=week; Tests #1-3 = physical function measures conducted by trained physiotherapists.

Table 2: Inclusion and Exclusion criteria

Inclusion Criteria:

- Adult patients ≥18 years old
- Mechanically ventilated ≤4 days with an expected additional 2 day ICU stay

Exclusion Criteria:

- Unable to ambulate independently prior to hospitalization (with or without a gait aid)
- Unable to follow simple commands at baseline
- Pregnancy
- Acute conditions impairing ability to walk or cycle (e.g., leg or foot fracture)
- Acute central condition or peripheral injury resulting in neuromuscular weakness (e.g., stroke, Guillian Barré syndrome)
- Primary generalized weakness of the central or peripheral nervous system (e.g., multiple sclerosis, myasthenia gravis, Parkinson's disease, amyotrophic lateral sclerosis)
- Temporary pacemaker wires
- Expected hospital mortality> 90%
- Cycling equipment unable to fit patient's body dimensions (e.g., leg amputation or obesity)
- Specific surgical exclusion as stipulated by attending
- >7 day ICU stay
- Physician declines
- Did not meet daily screening criteria (Table 3) during the first 4 days of mechanical ventilation

Table 3: Daily screening criteria for in-bed cycling

Research Sessions **will not occur** if **any** of the following conditions are present:

- Change in goals to palliative care
- Neuromuscular blocker within last 2 hours
- Active major haemorrhage from any site
- Known/suspected muscle inflammation (e.g., rhabdomyolysis)
- New pelvic, groin or extremity wound precluding lower extremity cycling.
- Acute peritonitis
- Platelet count <20,000/m^3
- Cardiac instability
    - Active myocardial ischaemia, or unstable/ uncontrolled arrhythmia per ICU team
    - HR <40 or >130 bpm within the last 2 hours
    - MAP <55 or >110 mmHg within the last 2 hours
    - ≥2 inotropes or vasopressors
    - Any increase in vasopressor/ inotrope within last 2 hours
- Persistent oxygen desaturation <85% despite repeated attempts to improve oxygenation (suctioning, bronchodilators, etc.) within the last 2 hours
- Femoral catheter *in situ*
- Severe agitation (Richmond Agitation and Sedation Scale[47] >2 [or equivalent]) within last 2 hours
- Team perception that in-bed cycling is not appropriate despite absence of above criteria (record reasons)

Table 4: Criteria to terminate in-bed cycling

Sessions will **stop** if:

- Sustained oxygen desaturation <88%, despite adjustments to FiO_2
- Heart rate
  - Low: 20 bpm less than lowest baseline value or 40 bpm (whichever is highest)
  - High: 20 bpm more than highest baseline value or 140 bpm (whichever is lowest)
- MAP <55 or >110 mmHg
- Marked ventilator dysynchrony not corrected by adjusting cycle off criteria
- Safety events:
  - Suspected new unstable / uncontrolled arrhythmia
  - Concern for myocardial ischaemia
  - Respiratory distress leading to symptoms of intolerable dyspnea
  - Unplanned extubation
  - Any of the following catheter or tube dislodgements
    - Catheters: Central venous, arterial, dialysis, or pulmonary artery catheter
    - Tubes: Orogastric, nasogastric, or percutaneous endoscopic gastrostomy
- ICU physician, patient or proxy requests termination of session

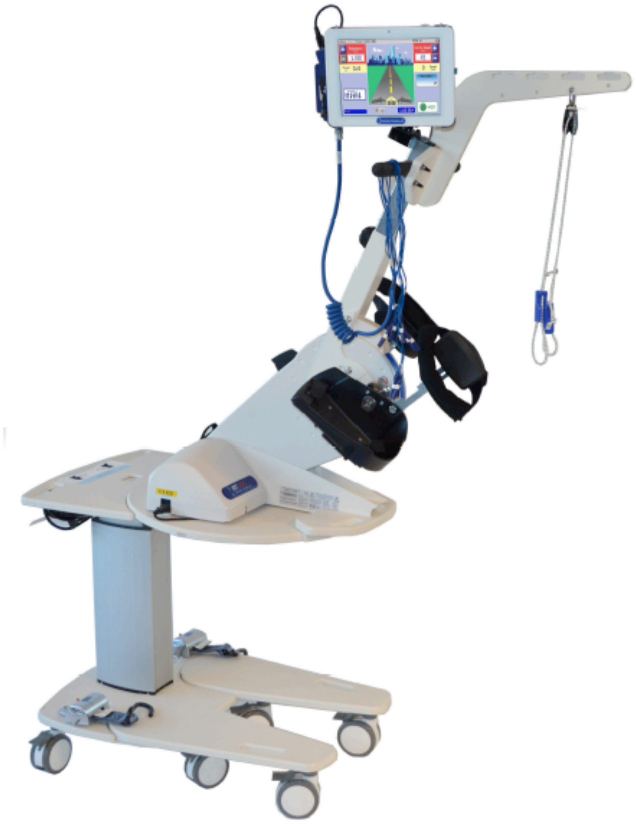

Figure 3: RT 300 Supine Cycle Ergometer

Table 5: Patient Variables

Patient Variables – collected at enrollment	Method of Collection	Measurement Scale
Age/sex	Chart review	Continuous/Binary
Comorbidities: Charlson* & Functional Indices**	Chart review	Ordinal
Functional Status Score for ICU(45)	Proxy interview	Continuous
Activities of Daily Living Scale[59]	Proxy interview	Continuous
Severity of illness: APACHE II***	Chart review	Continuous
Body mass index	Chart review	Continuous

Legend: **Charlson index: a score derived from 19 comorbidities, with an increased score reflecting increased 1-year mortality.*[82] ***Functional Index: an 18 diagnosis scale which uses physical function as the outcome, with increased score predicting decreased function.*[83, 84] ****APACHE II: An index of severity that takes into account age, pre-existing medical conditions, and 12 acute physiologic variables assessed during the first 24 hours of ICU. Scored on a scale from 0-71, with higher scores indicating more severe disease and increased short-term mortality.*[85]

Table 6: ICU-related Variables – Collected DAILY during ICU stay

ICU Variables	Method of Collection	Measurement Scale
ICU admission diagnosis	Chart review	Categorical
Benzodiazepine and opioid drug and dose	Chart review	Continuous
Sedation status	Chart review	Continuous
Neuromuscular blocker and steroid drug and dose	Chart review	Continuous
Insulin dose and blood glucose level	Chart review	Continuous
SOFA score*	Chart review	Continuous
Enteral nutrition – type, total volume, residual volume, calorie intake, protein intake	Chart review	Continuous
Parenteral nutrition – type, total volume, calorie intake, protein intake	Chart review	Continuous

Legend: ** SOFA: A validated composite score of 6 organ systems used to assess the severity of ICU organ dysfunction.*[86]

Table 7: Safety and Feasibility Outcomes

Objective	Outcome	Hypothesis	Comparison
**1**	**Safety**		
**1a**	**Adverse events**	The catheter or tube dislodgement event rate (Table 4) will not be different, or will be better (i.e., lower) than reported in the literature.	<4%[32]
**1b**	**Termination**	The *a priori* safety event rate (Table 4) will not be different, or will be better (i.e., lower) than reported in the literature.	0 to 4%[32]
**1c**	**Stability**	i) During cycling vital signs will not be different from pre-specified baseline levels (20% for HR, RR and BP [systolic or diastolic]).<br><br>ii) There is no change in variability among pre-, during, and post-cycling periods.	<20% change from baseline in HR, RR, and BP
**2**	**Feasibility**		
**2a**	**Consent**	Our consent rate will not be different, or will be better (i.e., higher) than existing literature.	70%[6]
**2b**	**Intervention delivery**	Our research session delivery rate will not be different, or will be better (i.e., higher) than existing literature.	80%[3]
**2c**	**Outcome measures**:	Our event rates for outcome measure ascertainment will be 80% or higher for each outcome measure, at each of the 3 time points (ICU awakening, ICU discharge, hospital discharge).	80%

Legend: *HR= heart rate; BP= blood pressure; RR=respiratory rate*

Table 8: Description of Physical Function Measures

Outcome	Instrument	Brief description of measure	Scale	Measurement Characteristics in ICU Patients
Leg and overall body strength	Manual muscle testing using MRC scale[57, 87]	The patient exerts a force against the examiner's resistance. Each muscle assessed on a 6-point MRC scale (0=no contraction; 5=contraction sustained against maximal resistance).	Ordinal	Inter-rater reliability (ICC[95%CI]): legs= 0.96[0.92 to 0.97]; overall body= 0.95 [0.92 to 0.97][57]
Quadriceps Leg Strength	Hand held dynamometer[57, 58]	The patient exerts a force against a small strain gauge that fits in the examiner's hand. Force measured in Kg and in Newtons.	Continuous	Inter-rater reliability (ICC[95%CI]) = 0.94 [0.90 to 0.97][58]
Grip Strength	Hand grip dynamometer[57]	The patient grasps a strain gauge measurement device. Force measured in Kg.	Continuous	Inter-rater reliability (ICC[95%CI]): R=0.93[0.86 to 0.97]; L=0.97[0.94 to 0.98][57]
Bed mobility and transfers	Functional Status Scale for ICU[37]	The patient attempts 5 bed mobility/ transfer tasks: rolling, supine to sit, sitting at edge of be, transfer from sit to stand, ambulation. Each item assessed on a scale from 0 (unable to perform) to 7 (independent).	Ordinal	Not established in ICU population
Activities of Daily Living	Katz Activities of Daily Living Scale[59]	The patient's ability to complete 6 tasks: bathing, dressing, toileting, feeding, continence, and bed mobility. A rater assesses whether the patient is dependent or independent according to pre-specified criteria.	Continuous	Not established in ICU population, however used in ICU RCT of early mobility[6]
Overall function	Physical Function Test for ICU[60]	The patient completes 4 tasks: sit to stand, marching on the spot, repeated bilateral shoulder flexion, and muscle strength assessment (knee extension, shoulder flexion).	Continuous	Inter-rater reliability (ICC [95% CI]): time marching on the spot= 1.0 [0.99 to 1.0]; shoulder flexion endurance time=1.0 [0.99 to 1.0][60]
Patient-rated function	Patient-Specific Functional Scale[65]	The patient rates their ability to complete a specific task on a scale from 0 (unable to perform activity) to 10 (able to perform activity at baseline level).	Continuous	Not tested in ICU population

Legend: *ICC= Intraclass correlation coefficient; 95% CI= 95% confidence interval;R=right; L=left.*

Table 9: Pilot implementation data from 185 patients receiving in-bed cycling at the Johns Hopkins Medical ICU[67] ***(data courtesy of Dale Needham, MD, PhD)***

Characteristic	N=185 patients
Age, mean (SD) years	56.3 (16.7)
Male, N (%)	104 (56.2)
Admission diagnosis, N (%)	
Acute respiratory failure	102 (55.1)
Sepsis	25 (13.5)
Gastrointestinal	42 (8.6)
Other	42 (22.7)
Received mechanical ventilation during ICU stay, N (%)	151 (81.6)
Ambulatory before ICU admission, N(%)	159 (85.9)
Time to first PT session from MICU admission, median [IQR] days	5 [2, 10]
Time to first cycling session, days, median [IQR] days	5 [2, 10]
Cycling as first PT session, N (%)	93 (50.3)

Legend: *PT= physiotherapy*

Table 10: Knowledge translation research receptors

Name of Research Receptor	Opportunity for knowledge translation, exchange, exploitation, commercialization, project social or economic impacts, projected impact on practice or policy.
Our research team – *Knowledge Translation*	• Refinement of inclusion/ exclusion criteria, consent, protocol, data collection, analysis
Clinicians at our study site- *Knowledge Translation*	• We will teach physicians, nurses, and therapists, about the long-term adverse effects of ICU bed rest and expose them to novel early rehabilitation approaches.
Researchers – *Knowledge Exchange and Translation*	• We will register our protocol on clinicaltrials.gov.<br>• We will translate our results through scientific presentations and peer-review publications.
Clinicians enrolling patients to the future RCT – *Knowledge Exchange*	• We will share educational materials (brochures, videos) of mechanically ventilated patients using cycle ergometry to help them understand its use in the ICU
Future research physiotherapists for the research program – *Knowledge Exchange*	• We will share our standard operating procedures and training materials for teaching clinicians about the safe and efficient set up and cleaning of the cycle ergometer.<br>• We will share our research protocol for use of the cycle ergometer.<br>• We will share educational materials (brochures, videos) of mechanically ventilated patients using cycle ergometry to help them understand its use in the ICU
Substitute decision makers, patients – *Knowledge Exchange*	• We will develop educational materials (brochures, videos, patient, family testimonials) of mechanically ventilated patients using cycle ergometry to help them understand its use in the ICU
ICUs with cycle ergometers – *Knowledge Exchange*	• We will share our standard operating procedures and training materials for teaching clinicians about the safe and efficient set up and cleaning of the cycle ergometer<br>• We will share our protocols for feeding and suggested physiological monitoring criteria
Clinical trialists – *Knowledge Exchange*	• We will share our standard operating procedures for use of the Electronic Clinical Trial Support System to streamline bedside data collection, communication, and maintain current research protocols
