# Supplementary material for: TryCYCLE: A Prospective Study of the Safety and Feasibility of Early In-Bed Cycling in Mechanically Ventilated Patients
Source: PLoS One. 2016 Dec 28;11(12):e0167561. doi: 10.1371/journal.pone.0167561 (PMC5193383; doi:10.1371/journal.pone.0167561)
Supplement: S1 Text — (DOCX) [file pone.0167561.s008.docx]

**Supplemental Author List 1**: **Canadian Critical Care Trials Group Membership as of November 2016.**

Neill Adhikari, Stéphane P. Ahern, Aemal Akhtar, Martin Albert, Victoria Alcuaz, Waleed Alhazzani, Eyad Althenayan, Shelley Anderson-White, Natalie Anton, Patrick Archambault, Erika Arseneau, Pierre Aslanian, Fiona Auld, Sean M. Bagshaw, Andrew Baker, Ian Ball, Jane Batt, William Beaubien, Emilie Belley-Cote, Tracey Bentall, Virginie Bolduc, Karen J. Bosma, Stephane Bourque, Gord Boyd, Laurent Brochard, Karen E. Burns, Lisa D. Burry, Jeff Burzynski, Eileen Campbell, Josie Campisi, Elaine Carbonneau, François Carrier, Alexandre Cavayas, Emmanuel Charbonney, Michaël Chassé, Annick Chatillon, Karen Choong, Michael D. Christian, France Clarke, Lucy Clayton, Bryan Coburn, Anna Colucci, Deborah Cook, Joseph Dahine, Craig Dale, Nick Daneman, Frederick D'Aragon, Stephane Delisle, Sonny Dhanani, Joanna Dionne, Peter Dodek, James Downar, John Drover, Karen Dryden-Palmer, Mark Duffett, Mariana Dumitrascu, Christopher Ellis, Guillaume Emeriaud, Shane English, Eddy Fan, Catherine Farrell, Niall Ferguson, Patricia Fontela, Denise Foster, Jennifer Foster, Robert Fowler, Alison Fox-Robichaud, Charles Francoeur, Andreas Freitag, Anne Julie Frenette, Jan Friedrich, Kelly Fusco, Gonzalo Garcia Guerra, Allan Garland, Elaine Gilfoyle, Martin Girard, Ewan Goligher, Brigette Gomes, Milagros Gonzales, Ronald Gottesman, Robert Green, Donald Griesdale, Anne-Marie Guerguerian, Edita Hajdini, Adnan Haj-Moustafa, Richard Hall, Dallas Hall, Cindy Hamielec, BJ Hancock, Lori Hand, Paul C. Hebert, Ahmed Hegazy, Margaret Herridge, Mark Heule, Shawn Hicks, Miranda Hunt, James Hutchison, Angela Jerath, Ari Joffe, Jennie Johnstone, Gwynne Jones, Philippe Jouvet, Caroline Just, Salmaan Kanji, Tim Karachi, Oliver Karam, Constantine Karvellas, Brian Kavanagh, Atsushi Kawaguchi, Rachel Khadaroo, Michelle Kho, Kosar Khwaja, Niranjan Kissoon, Lisa Klotz, Barbara Kosky, Andreas Kramer, Arnold Kristof, Kristina Krmpotic, Paul Kubes, Anand Kumar, Demetrios James Kutsogiannis, Jacques Lacroix, Manoj Lalu, Francois Lamontagne, Heather Langlois, Francois Lauzier, Madelyn Law, Nicole LeBlanc, Liane Leclair, Murdoch Leeies, Caroline Leger, Aleksandra Leligdowicz, Patricia Liaw, Osama Loubani, Meghan MacKenzie, Sheldon Magder, Nicole Marinoff, John C. Marshall, Nicole Marten, Claudio Martin, Amanda Martyniuk, David Maslove, Marie-Hélène Masse, Andrea Matte, Victoria McCredie, Ellen McDonald, Lauralyn McIntyre, James McNally, Maureen Meade, Sangeeta Mehta, Nav Mehta, Tina Mele, Asher Mendelson, Kusum Menon, Tina Millen, Tamara Mitterer, Rohit Mohindra, Alexander Molloy, Haifa Mtaweh, Srinivas Murthy, John Muscedere, Dave Nagpal, Katie O’Hearn, Nicole O'Callaghan, Simon Oczkowski, Dawn Opgenorth, Daniel Ovakim, Joe Pagliarello, Elizabeth Papathanassoglou, Melissa Parker, Jeanna Parsons Leigh, Nicole Poitras, Sandra Pong, Rebecca Porteous, Oleksa Rewa, Asgar Rishu, Leena Rizvi, Bram Rochwerg, Louise Rose, Stephanie Rotella, Kurtis Salway, Marlene Santos, Aimee Sarti, Lois Saunders, Damon C. Scales, Nathan Scales, Andrew Seely, Mypinder Sekhon, Sumesh Shah, Jason Shahin, Michael Sharpe, Ilona Shemyakina, Cathy Sheppard, Stephanie Sibley, Robit Singal, Jeffrey M. Singh, Tasnim Sinuff, Yoanna Skrobik, Orla Smith, Henry T. Stelfox, Maude St-Onge, Asumi Sugiura, Eric Sy, Benjamin Tam, Samantha Taylor, Kelly Thompson, Alan Tinmouth, Bharath Kumar Tirupakuzhi Vijayaraghava, Jennifer Tsang, Marisa Tucci, Alexis Turgeon, Amanda van Beinum, Judith Van Huyse, Stefannie Vorona, Ron Wald, Irene Watpool, Matthew Weiss, David Wensley, Elizabeth Wilcox, David Williamson, Elisa Wilson, Brent Winston, Davinia E. Withington, Lynette Wohlgemuth, Gordon Wood, Hannah Wunsch, Lauren Zarnett, Ryan Zarychanski, David Zygun, and Nicole Zytaruk.
